# Faecal immunochemical tests (FIT) versus colonoscopy for surveillance after screening and polypectomy: a diagnostic accuracy and cost-effectiveness study

**DOI:** 10.1136/gutjnl-2018-317297

**Published:** 2018-12-11

**Authors:** Amanda J Cross, Kate Wooldrage, Emma C Robbins, Ines Kralj-Hans, Eilidh MacRae, Carolyn Piggott, Iain Stenson, Aaron Prendergast, Bhavita Patel, Kevin Pack, Rosemary Howe, Nicholas Swart, Julia Snowball, Stephen W Duffy, Stephen Morris, Christian von Wagner, Stephen P Halloran, Wendy S Atkin

**Affiliations:** 1 Cancer Screening and Prevention Research Group (CSPRG), Department of Surgery and Cancer, Imperial College London, London, UK; 2 Bowel Cancer Screening Programme Southern Hub, Guildford, UK; 3 Department of Applied Health Research, University College London, London, UK; 4 Centre for Cancer Prevention, Wolfson Institute of Preventive Medicine, Queen Mary University, London, UK; 5 Research Department of Behavioural Science and Health, University College London, London, UK; 6 Faculty of Health and Medical Sciences, University of Surrey, Guildford, UK

**Keywords:** adenoma, colorectal cancer, surveillance, stool markers, colonoscopy

## Abstract

**Objective:**

The English Bowel Cancer Screening Programme (BCSP) recommends 3 yearly colonoscopy surveillance for patients at intermediate risk of colorectal cancer (CRC) postpolypectomy (those with three to four small adenomas or one ≥10 mm). We investigated whether faecal immunochemical tests (FITs) could reduce surveillance burden on patients and endoscopy services.

**Design:**

Intermediate-risk patients (60–72 years) recommended 3 yearly surveillance were recruited within the BCSP (January 2012–December 2013). FITs were offered at 1, 2 and 3 years postpolypectomy. Invitees consenting and returning a year 1 FIT were included. Participants testing positive (haemoglobin ≥40 µg/g) at years one or two were offered colonoscopy early; all others were offered colonoscopy at 3 years. Diagnostic accuracy for CRC and advanced adenomas (AAs) was estimated considering multiple tests and thresholds. We calculated incremental costs per additional AA and CRC detected by colonoscopy versus FIT surveillance.

**Results:**

74% (5938/8009) of invitees were included in our study having participated at year 1. Of these, 97% returned FITs at years 2 and 3. Three-year cumulative positivity was 13% at the 40 µg/g haemoglobin threshold and 29% at 10 µg/g. 29 participants were diagnosed with CRC and 446 with AAs. Three-year programme sensitivities for CRC and AAs were, respectively, 59% and 33% at 40 µg/g, and 72% and 57% at 10 µg/g. Incremental costs per additional AA and CRC detected by colonoscopy versus FIT (40 µg/g) surveillance were £7354 and £180 778, respectively.

**Conclusions:**

Replacing 3 yearly colonoscopy surveillance in intermediate-risk patients with annual FIT could reduce colonoscopies by 71%, significantly cut costs but could miss 30%–40% of CRCs and 40%–70% of AAs.

**Trial registration number:**

ISRCTN18040196; Results.

**Significance of this study**

**What is already known on this subject?**

- Colonoscopy surveillance is recommended for patients who remain at increased risk of colorectal cancer (CRC) following polypectomy.
- Low-risk, intermediate-risk and high-risk groups are defined according to baseline adenoma characteristics.
- Intermediate-risk patients (with three to four adenomas <10 mm or at least one ≥10 mm) are recommended 3 yearly colonoscopy surveillance.
- The burden of postpolypectomy surveillance is substantial for both patients and endoscopy services.
- The faecal immunochemical test (FIT), widely used for CRC screening, may be an effective alternative to colonoscopy surveillance, but few data are available on FIT in surveillance settings.

**What are the new findings?**

- Among intermediate-risk patients, annual FIT was well accepted with initial consent and FIT return of 74% and subsequent FIT return of 97%.
- Positivity increased at lower FIT thresholds. At the lowest threshold of 10 µg haemoglobin per gram (10 µg/g) faeces, 3-year cumulative positivity was 29%, compared with 13% at 40 µg/g.
- Sensitivities increased at lower thresholds. Three-year programme sensitivities for CRC and advanced adenomas (AAs) were, respectively, 59% and 33% at 40 µg/g and 72% and 57% at 10 µg/g.
- The incremental cost-effectiveness of colonoscopy versus FIT surveillance was £7354 per additional AA detected and £180 778 per additional CRC detected.

**Significance of this study**

**How might it impact on clinical practice in the foreseeable future?**

- Replacing 3 yearly colonoscopy surveillance in intermediate-risk patients with annual FIT could reduce numbers of colonoscopies by ≥70% and produce significant cost savings.
- Annual FIT could, however, miss 30%–40% of CRCs and 40%–70% of AAs, depending on the threshold used.
- Further economic analyses and longer term studies are warranted to define a clear role for FIT in surveillance.

## Introduction

Most colorectal cancers (CRCs) arise from adenomas,^1 2^ and adenoma removal reduces CRC incidence and mortality.^3–6^ Some patients remain at increased risk of CRC postpolypectomy,^7 8^ and national guidelines recommend surveillance by colonoscopy.^9–12^ The UK surveillance guidelines recommend different strategies for patients at low risk, intermediate risk and high risk.^9^ Surveillance is not recommended for low-risk patients (with one to two adenomas <10 mm), while 3 yearly surveillance is recommended for intermediate-risk patients (with three to four adenomas <10 mm or at least one ≥10 mm). High-risk patients (with five or more adenomas <10 mm, or three or more adenomas with at least one ≥10 mm) are recommended colonoscopy at 1 year before commencing 3 yearly surveillance.

Colonoscopy surveillance is associated with reduced CRC incidence^8^; however, this procedure can have serious complications and be uncomfortable and anxiety inducing.^13 14^ Colonoscopy surveillance places great demand on endoscopy services, accounting for 20% of colonoscopies in the UK.^15^ Further increases in endoscopy demand due to widespread implementation of CRC screening and higher rates of primary care referrals for suspected CRC^16^ give grounds for finding alternative surveillance methods.

The faecal immunochemical test (FIT) may be an alternative. Like the guaiac faecal occult blood test (gFOBT), FIT detects occult blood in faeces, although it detects globin rather than haem, making it more specific for human blood.^17^ FIT is replacing gFOBT in CRC screening programmes because of its ease of use, increased uptake, quantitative analysis and greater sensitivity for advanced colorectal neoplasia (ACN).^18 19^ The English Bowel Cancer Screening Programme (BCSP) is planning to introduce FIT in 2018.^20^

FIT has been extensively evaluated for screening, with sensitivity estimates of a single low-threshold FIT for CRC approaching 90%.^21^ There are few studies on FIT for surveillance, and these reported sensitivities for CRC of 70%–100%, although they included patients attending surveillance for personal or family histories of CRC.^22–27^ We therefore developed the ‘FIT for Follow-Up’ study to investigate the potential utility of FIT for postpolypectomy surveillance, looking specifically at patients deemed at intermediate-risk following screening within the BCSP. We focused on intermediate-risk patients as they account for most surveillance colonoscopies in the UK.^8 28^ Here we report the diagnostic accuracy of annual FIT for CRC and advanced adenomas (AAs) over 3 years versus 3 yearly colonoscopy, alongside estimates of cost-effectiveness.

## Methods

### Study design and participants

Individuals aged 60–72 years were considered eligible if deemed at intermediate risk of CRC following colonoscopy performed <1 year previously in the BCSP for a positive gFOBT and scheduled to undergo 3 yearly colonoscopy surveillance. Individuals with >1 baseline colonic examination were excluded to prevent overinvestigation from examinations performed early due to positive FIT.

Potentially eligible individuals were identified by National Health Service (NHS) Digital using the Bowel Cancer Screening System (BCSS). Information on these individuals was sent in encrypted form by NHS Digital to the BCSP Southern Hub, one of five BCSP hubs across England. The hubs send invitations, mail and process faecal occult blood tests and record test results for their regional population. Although all five hubs were involved in this study, the Southern Hub coordinated all of these functions using a bespoke electronic patient management system (PMS). Each hub is associated with screening centres; 64 screening centres were involved in this study.

From January 2012 to December 2013, the Southern Hub sent invitations to consecutive individuals meeting the eligibility criteria, together with a participant information sheet, consent form and FIT kit. The kit contained a FIT device (OC-AUTO Sampling Bottle 3, Eiken Chemical Co Ltd, Japan), instructions, a plastic zip-lock bag and a prepaid envelope to return the kit to the Southern Hub.

Individuals who consented and returned an analysable FIT were included. Kits received >10 days after sample collection were not accepted. Samples were refrigerated on receipt and analysed within 1 week using the OC-Sensor DIANA analyser and new formulation sample buffer^29^ (Eiken Chemical Co Ltd, supplied by MAST Group Ltd, UK), according to manufacturer instructions. Results were entered on to the PMS. Repeat kits were dispatched to individuals who had lost theirs or had inadequate or spoilt samples.

We initially used a positivity threshold of 20 µg haemoglobin per gram (µg/g) faeces. At this threshold, the positivity rate of the first 65 kits was 9.2%, higher than expected from the literature.^30 31^ We were concerned that if this rate was sustained, too many participants would be offered early colonic examinations and no further FITs, putting pressure on endoscopy services and affecting our ability to model FIT performance at different thresholds. The threshold was therefore raised to 40 µg/g. Of the six kits classed as positive using the 20 µg/g threshold, three had readings below 40 µg/g. These were not retrospectively reclassified as negative following the threshold change.

Participants were offered FITs at 1, 2 and 3 years postpolypectomy. Participants testing positive at years 1 or 2 were offered early colonic examination and no further FITs. Participants not attending the early examination were invited to the 3-year examination, as per UK guidelines.^9^ Participants testing negative at years 1 or 2 were offered further FITs, as were those who did not complete their year 2 FIT. All participants offered a year 3 FIT were invited to the routine 3-year examination. Findings at colonic examination (early or 3 year) were the reference standard against which we measured the diagnostic accuracy of FIT.

The default colonic examination was colonoscopy performed by accredited endoscopists, the ‘gold-standard’ for detection of colorectal lesions.^32^ CT colonography was an alternative for participants unfit for colonoscopy. Endoscopy findings were not known by those interpreting FIT results; however, endoscopists may have been aware when examinations were for positive FIT results.

### Data collection and management

FIT results were reported as positive or negative to participants and their general practitioners. For participants testing positive at years 1 or 2, affiliated screening centres were informed of their participation in the study, positive FIT result and that they should be offered an early colonic examination. Early examinations were organised by Specialist Screening Practitioners and administrators at the screening centres.

Colonic examination and pathology reports from early and 3-year examinations were obtained from screening centres. Patient, procedural and polyp data from these reports were entered on to the PMS using standard operating procedures. For some participants (n=28), we could not obtain all reports from screening centres, and the BCSS was used as a supplementary data source. Some participants received examinations at hospitals outside the BCSP. When made aware of this, usually by participants contacting the BCSP, we attempted to retrieve the reports; reports for 42 participants were retrieved in this way.

Colonic examinations occurring between consent date and 18 months after scheduled 3-year examination were included. Participants with examinations known to be incomplete or have poor visualisation (n=32) were not included as having examinations performed, as we could not determine accurately whether colorectal lesions were present. Participants may have undergone a combination of colonic examinations or surgery; all procedures were considered when defining outcomes of CRC and AAs. Cancer diagnoses to the end of 2014 were obtained from the English cancer registry. Participants with no record of a colonic examination occurring during the study but found to have CRC in registry data (n=2) were included as having examinations with CRC detected.

Polyp size was defined by the largest diameter reported on colonic examination, pathology or surgical reports. Adenomas ≥10 mm, with high-grade dysplasia, villous or tubulovillous histology were defined as AAs. We defined CRC sites by the International Classification of Diseases 10th revision, using codes C18–C20. We coded CRC morphologies using International Classification of Diseases for Oncology 2nd edition codes, including 8070/3, 8140/3, 8210/3, 8240/3, 8244/3, 8263/3, 8480/3 and 8481/3. We included all histological subtypes of CRC that arose in our dataset in our main analysis as surveillance programmes would ideally detect all cancers in the lower GI tract.

### Statistical analysis

Our sample size calculation was based on an estimated relative sensitivity for ACN of three annual FITs versus 3 yearly colonoscopy. Assuming an ACN prevalence of 2.5%, relative FIT sensitivity of 75% and 40% compliance with all tests, 72 cases, 2881 adherent participants and 8000 invitees were required to provide sensitivity estimates with 95% CIs within ±10% and margin of error of ±10%.

We calculated rates of FIT return and positivity and diagnostic yields of CRC and AAs in participants who underwent colonic examination following positive FIT. We analysed the sensitivity, specificity, positive predictive value (PPV) and negative predictive value (NPV) of FIT for CRC and AAs at the 40 µg/g threshold. For the analyses of diagnostic accuracy for AAs, we excluded participants with CRC as we expected their inclusion would lead to biased estimates because FIT is more sensitive for CRC than AAs.^33^

We estimated diagnostic accuracy at lower thresholds (10 µg/g, 20 µg/g and 30 µg/g) and with multiple tests, assuming that any ACN detected was present from year 1, remaining unchanged over successive years in the absence of colonic examination. Considering thresholds below 40 µg/g, we assumed that participants with faecal haemoglobin levels above the particular threshold underwent hypothetical early examinations, which detected what was actually found later on. Results from subsequent FITs were excluded from these analyses.

For analyses with multiple tests, we conducted cumulative test and programme analyses. For cumulative test analyses, participants were included if they completed the designated number of FITs (two for two-test analyses; three for three-test analyses) or tested positive with a previous FIT. All study participants were included in programme analyses; in two-test analyses, participants were ‘positive’ if they tested positive with either of their first two FITs, while in three-test analyses, participants were ‘positive’ if they tested positive with any FIT.

We conducted subgroup analyses by sex and age (≤65 years and >65 years) at invitation date. In sensitivity analyses, we examined effects on diagnostic accuracy estimates of excluding non-adenocarcinomas (squamous cell carcinomas and neuroendocrine tumours); these are rare cancers that account for <5% of CRCs.^34 35^ Further sensitivity analyses involved excluding participants with colonic examinations of unknown quality and those with IBD.

We assessed 3-year surveillance costs of annual FIT with colonoscopy in positive cases versus 3 yearly colonoscopy, calculating total costs and mean costs per participant. Cost-effectiveness was presented as incremental costs per additional AA and CRC detected by colonoscopy versus FIT surveillance. We estimated the budget impact of replacing colonoscopy with FIT surveillance nationally over a screening cycle (supplementary material: Economic analysis of faecal immunochemical test versus colonoscopy surveillance).

Analyses were performed in Stata/IC V.13. The protocol is online.^36^

## Results

**Figure 1:**
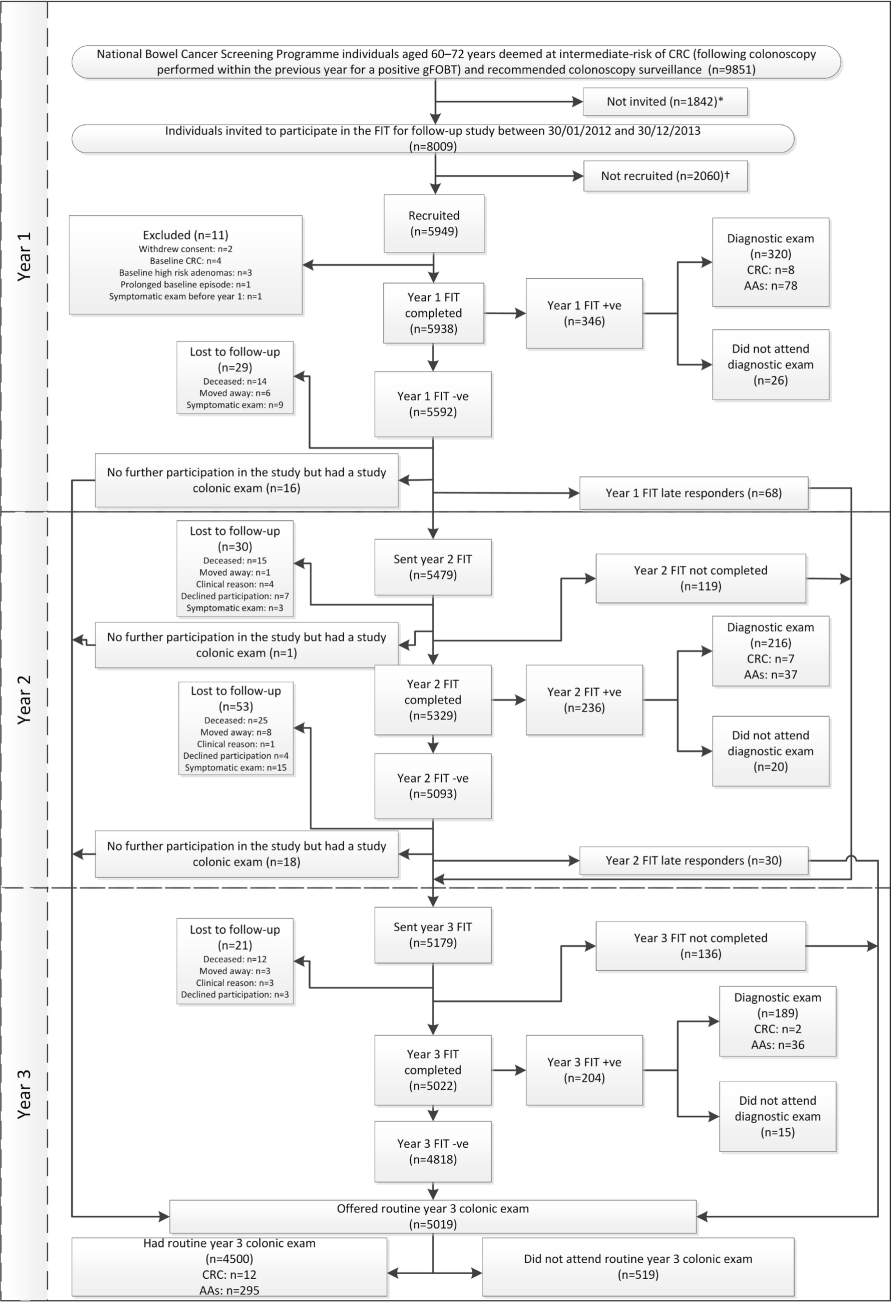
Participant flow diagram from invitation through to year 3 colonic examination. *Two hundred and ninety-five potentially eligible individuals were not invited as they were excluded after the eligibility assessment (186 in order to prevent over-investigation as they had already undergone more than one colonoscopy and 109 due to informed dissent, clinical reasons, death or emigration) and a further 1547 individuals were not invited as the sample size of 8000 had already been met.†Two thousand and fifty-five individuals were not recruited due to lack of consent; one consented but did not return their FIT; and four consented but returned a FIT that could not be analysed. AAs, advanced adenomas; CRC, colorectal cancer; FIT, faecal immunochemical test; gFOBT, guaiac faecal occult blood test.

There were 9851 potentially eligible individuals. Of these, we excluded 186 with multiple baseline examinations and 109 due to informed dissent, clinical reasons, death or emigration. An additional 1547 individuals were not invited as the sample size of 8000 had been met. In total, 8009 individuals were invited (figure 1). The proportions of invitees aged ≤65 years and >65 years were, respectively, 49.3% and 50.7%; 34.7% were women (table 1).

**Table 1:** Baseline characteristics of invited individuals by participation

Sex	Age at invitation date (years)	Invited		Participants*		Non-participants*		**P value**†
		N	(%)‡	n	(%)	n	(%)	
**All**	**All ages**	8009	(100.0)	5938	(74.1)	2071	(25.9)	
	**≤65**	3950	(49.3)	2877	(72.8)	1073	(27.2)	0.008
	**>65**	4059	(50.7)	3061	(75.4)	998	(24.6)	
**Men**	**All ages**	5228	(65.3)	3892	(74.4)	1336	(25.6)	
	**≤65**	2634	(32.9)	1901	(72.2)	733	(27.8)	<0.001
	**>65**	2594	(32.4)	1991	(76.8)	603	(23.2)	
**Women**	**All ages**	2781	(34.7)	2046	(73.6)	735	(26.4)	
	**≤65**	1316	(16.4)	976	(74.2)	340	(25.8)	0.501
	**>65**	1465	(18.3)	1070	(73.0)	395	(27.0)	

*Participants were individuals who gave consent, returned an analysable FIT at year 1 and did not subsequently withdraw from the study.

†P values are for the comparison of age at invitation date for participants and non-participants, overall and by sex.

‡Percentage of the total invited cohort (8009).

Of those invited, 2060 were not recruited: 2055 did not consent and five consented but did not return an analysable FIT. A further 11 were excluded due to withdrawal of consent, baseline CRC, baseline high-risk adenomas, prolonged baseline episode or symptomatic exam before year 1 (figure 1). Therefore, 5938 of 8009 (74.1%) invitees were recruited, having consented and returned an analysable FIT (table 1). Return of FIT was 97% at years 2 and 3 (table 2). Participation was similar among men and women and across age groups (online supplementary tables 1 and 2).

10.1136/gutjnl-2018-317297.supp1 Supplementary file 1

**Table 2:** Uptake, positivity rate and diagnostic yield of the faecal immunochemical test (FIT) at years 1, 2 and 3 and over all 3 years

	Uptake			Positivity rate		Colonic exam performed				Diagnostic yield*			
	Invited	**Completed FIT test**†		**Tested positive**‡		**Any exam**§		**Colonoscopy**¶		Colorectal cancer		Advanced adenomas**	
**Year**	n	n	(%)	n	(%)	n	(%)	n	(%)	n	(%)	n	(%)
1	8009	5938	(74.1)	346††	(5.8)	320	(92.5)	317	(99.1)	8	(2.5)	78	(24.4)
2	5479	5329	(97.3)	236	(4.4)	216	(91.5)	212	(98.1)	7	(3.2)	37	(17.1)
3	5179	5022	(97.0)	204	(4.1)	189	(92.6)	184	(97.4)	2	(1.1)	36	(19.0)
Cumulative	8009	5938	(74.1)	786‡‡	(13.2)	725	(92.2)	713	(98.3)	17	(2.3)	151	(20.8)
**Routine year 3 colonic exam**						4500		4420	(98.2)	12	(0.3)	295	(6.6)
**Entire study findings**						5225		5133	(98.2)	29	(0.6)	446	(8.5)

*Diagnostic yield in participants who underwent colonic examination.

†Participants who gave consent, returned an analysable FIT at year 1 and did not subsequently withdraw from the study.

‡Percentages calculated using the number of participants who completed FIT as the denominator. In the pilot study, a threshold of 20 µg haemoglobin (Hb)/g faeces was used to denote test positivity. The positivity threshold used in the rest of the study was 40 µg Hb/g faeces.

§Participants who underwent colonoscopy or CT colonography. Percentages calculated using the number of FIT positive participants as the denominator.

¶Participants who had a colonoscopy. Percentages calculated using the number of participants who underwent colonic examination as the denominator.

**Advanced adenomas were defined as adenomas ≥10 mm, with villous or tubulovillous histology, or high-grade dysplasia.

††Three participants tested positive at year 1 during the pilot study based on a threshold of 20 µg Hb/g. They are included as FIT positive in this table even though their faecal haemoglobin levels were lower than the 40 µg Hb/g threshold used in the rest of the study.

‡‡Participants who were FIT positive with any FIT, regardless of whether they had completed all FITs that they were offered.

FIT positivity decreased from 5.8% to 4.1% over years 1–3. Cumulative positivity over 3 years was 13.2% (table 2) and greater in men than women (14.7% vs 10.4%) and in participants aged >65 years than those ≤65 years (14.3% vs 12.1%) (online supplementary tables 1 and 2).

Colonic examinations were performed at some time during the study in 5225 of 5938 (88.0%) participants, yielding CRC in 29 (0.6%) and AAs in 446 (8.5%) (table 2). There were six participants with CRC and AAs. Of the 29 CRCs, 15 were proximal and 14 distal to the splenic flexure, and 26 were adenocarcinomas. Of the 26 CRCs with stage information, 10 were stage III or IV (online supplementary table 3).

Over 3 years, 725 (12.2%) participants underwent colonic examination following positive FIT, finding CRC in 17 (2.3%) and AAs in 151 (20.8%) (table 2). FIT therefore identified 58.6% of participants with CRC (17/29) and 33.9% of participants with AAs (151/446) over 3 years at a threshold of 40 µg/g. Diagnostic yields at years 1, 2 and 3 were, respectively, 2.5%, 3.2% and 1.1% for CRC, and 24.4%, 17.1% and 19.0% for AAs (table 2).

FIT positivity was higher at lower thresholds. In programme analysis, 3-year positivity was 13.2% at 40 µg/g, and 28.8% at 10 µg/g (table 3). Sensitivities for CRC increased while specificities decreased at lower thresholds and with multiple tests. Sensitivities and specificities of the first FIT were, respectively, 27.6% and 94.1% at 40 µg/g, and 51.7% and 86.2% at 10 µg/g (table 4). Over 3 years, programme sensitivities were 58.6% at 40 µg/g and 72.4% at 10 µg/g, and programme specificities were 86.4% at 40 µg/g and 71.1% at 10 µg/g. Similar patterns were observed in cumulative test analyses (table 4). Diagnostic accuracy estimates at 30 µg/g and 20 µg/g are shown in online supplementary table 4.

**Table 3:** Positivity rate of the faecal immunochemical test (FIT) at different thresholds in participants who completed one, two, or three tests

FIT threshold (µg/g)	Test		All		
			Completed test*	Tested positive	
			n	n	(%)
**40**		First	5938	343†	(5.8)
		Second‡	5459	245	(4.5)
		Third§	4892	195	(4.0)
	**Over two tests**	**CTA¶**	5802	588	(10.1)
		PA**	5938	588	(9.9)
	**Over three tests**	**CTA**††	5480	783	(14.3)
		**PA**‡‡	5938	783	(13.2)
**30**		First	5938	415	(7.0)
		Second‡	5393	293	(5.4)
		Third§	4785	238	(5.0)
	**Over two tests**	**CTA**¶	5808	708	(12.2)
		PA**	5938	708	(11.9)
	**Over three tests**	**CTA**†**†**	5493	946	(17.2)
		**PA**‡‡	5938	946	(15.9)
**20**		First	5938	545	(9.2)
		Second‡	5274	357	(6.8)
		Third§	4616	291	(6.3)
	**Over two tests**	**CTA**¶	5819	902	(15.5)
		PA**	5938	902	(15.2)
	**Over three tests**	**CTA**†**†**	5518	1193	(21.6)
		**PA‡‡**	5938	1193	(20.1)
**10**		First	5938	841	(14.2)
		Second‡	4986	487	(9.8)
		Third§	4225	384	(9.1)
	**Over two tests**	**CTA¶**	5857	1328	(22.8)
		PA**	5938	1328	(22.4)
	**Over three tests**	**CTA**†**†**	5553	1712	(30.8)
		**PA‡‡**	5938	1712	(28.8)

*Participants who tested positive at a given threshold at year 1 or 2 were excluded from subsequent analyses.

†Three participants tested positive at year 1 during the pilot study based on faecal haemoglobin levels higher than 20 µg haemoglobin (Hb)/g but lower than the 40 µg Hb/g threshold used in the rest of the study. In this table, they only appear as FIT positive if their faecal haemoglobin levels met the stated thresholds.

‡Includes participants who completed their second FIT, either at year 2 or 3.

§Includes participants who completed their third FIT.

¶Includes participants who completed at least two FITs or who tested positive at year 1. Participants were classed as positive if they tested positive with either of their first two FITs.

**Includes participants who completed at least one FIT. Participants were classed as positive if they tested positive with either of their first two FITs.

††Includes participants who completed all three FITs or who tested positive with any FIT. Participants were classed as positive if they tested positive with any FIT.

‡‡Includes participants who completed at least one FIT. Participants were classed as positive if they tested positive with any FIT.

CTA, cumulative test analysis; PA, programme analysis.

**Table 4:** Sensitivity, specificity, positive predictive value (PPV) and negative predictive value (NPV) of the faecal immunochemical test (FIT) at thresholds of 40 µg/g and 10 µg/g for colorectal cancer in participants who completed one, two, or three tests and underwent colonic examination

FIT threshold (µg/g)	Test		Completed test*	Participants with colorectal cancer		Participants without colorectal cancer		Sensitivity (95% CI)	Specificity (95% CI)	PPV (95% CI)	NPV (95% CI)
			n	TP	FN	FP	TN	%	%	%	%
**40**		First	5225	8	21	309	4887	27.6 (12.7 to 47.2)	94.1 (93.4 to 94.7)	2.5 (1.1 to 4.9)	99.6 (99.3 to 99.7)
		Second†	4864	7	9	218	4630	43.8 (19.8 to 70.1)	95.5 (94.9 to 96.1)	3.1 (1.3 to 6.3)	99.8 (99.6 to 99.9)
		Third‡	4448	2	5	178	4263	28.6 (3.7 to 71.0)	96.0 (95.4 to 96.5)	1.1 (0.1 to 4.0)	99.9 (99.7 to 100.0)
	**Over two tests**	**CTA**§	5181	15	9	527	4630	62.5 (40.6 to 81.2)	89.8 (88.9 to 90.6)	2.8 (1.6 to 4.5)	99.8 (99.6 to 99.9)
		**PA**¶	5225	15	14	527	4669	51.7 (32.5 to 70.6)	89.9 (89.0 to 90.7)	2.8 (1.6 to 4.5)	99.7 (99.5 to 99.8)
	**Over three tests**	CTA**	4990	17	5	705	4263	77.3 (54.6 to 92.2)	85.8 (84.8 to 86.8)	2.4 (1.4 to 3.7)	99.9 (99.7 to 100.0)
		**PA**††	5225	17	12	705	4491	58.6 (38.9 to 76.5)	86.4 (85.5 to 87.4)	2.4 (1.4 to 3.7)	99.7 (99.5 to 99.9)
**10**		First	5225	15	14	718	4478	51.7 (32.5 to 70.6)	86.2 (85.2 to 87.1)	2.0 (1.1 to 3.4)	99.7 (99.5 to 99.8)
		Second†	4458	5	6	437	4010	45.5 (16.7 to 76.6)	90.2 (89.3 to 91.0)	1.1 (0.4 to 2.6)	99.9 (99.7 to 99.9)
		Third‡	3853	1	3	345	3504	25.0 (0.6 to 80.6)	91.0 (90.1 to 91.9)	0.3 (0.0 to 1.6)	99.9 (99.8 to 100.0)
	**Over two tests**	**CTA**§	5191	20	6	1155	4010	76.9 (56.4 to 91.0)	77.6 (76.5 to 78.8)	1.7 (1.0 to 2.6)	99.9 (99.7 to 99.9)
		**PA**¶	5225	20	9	1155	4041	69.0 (49.2 to 84.7)	77.8 (76.6 to 78.9)	1.7 (1.0 to 2.6)	99.8 (99.6 to 99.9)
	**Over three tests**	CTA**	5028	21	3	1500	3504	87.5 (67.6 to 97.3)	70.0 (68.7 to 71.3)	1.4 (0.9 to 2.1)	99.9 (99.8 to 100.0)
		**PA**††	5225	21	8	1500	3696	72.4 (52.8 to 87.3)	71.1 (69.9 to 72.4)	1.4 (0.9 to 2.1)	99.8 (99.6 to 99.9)

*Participants who tested positive at a given threshold at year 1 or 2 were excluded from subsequent analyses.

†Includes participants who completed their second FIT, either at year 2 or 3.

‡Includes participants who completed their third FIT.

§Includes participants who completed at least two FITs or who tested positive at year 1. Participants were classed as positive if they tested positive with either of their first two FITs.

¶Includes participants who completed at least one FIT. Participants were classed as positive if they tested positive with either of their first two FITs.

**Includes participants who completed all three FITs or who tested positive with any FIT. Participants were classed as positive if they tested positive with any FIT.

††Includes participants who completed at least one FIT. Participants were classed as positive if they tested positive with any FIT.

CTA, cumulative test analysis; FN, false negative; FP, false positive; PA, programme analysis; TN, true negative; TP, true positive.

At lower thresholds, greater proportions of FIT-detected CRCs were found at year 1 (71.4% at 10 µg/g versus 47.1% at 40 µg/g) (data not shown). PPVs for CRC were smaller at lower thresholds and with multiple tests. In programme analysis after 3 years, PPVs were 2.4% at 40 µg/g and 1.4% at 10 µg/g. NPVs fell between 99.6% and 99.9% for all thresholds and numbers of tests (table 4).

Subgroup analyses by sex and age were underpowered; however, sensitivities were generally higher in older (>65 years) participants, while specificities were generally higher in younger (≤65 years) participants and women (online supplementary tables 5 and 6).

FIT was less sensitive but more specific for AAs than CRC. Sensitivities for AAs increased and specificities decreased at lower thresholds and with multiple tests. Three-year programme sensitivities for AAs were 33.4% at 40 µg/g and 56.6% at 10 µg/g, and programme specificities were 88.3% at 40 µg/g and 73.7% at 10 µg/g (table 5). PPVs were higher and NPVs lower for AAs than for CRC (table 5).

**Table 5:** Sensitivity, specificity, positive predictive value (PPV) and negative predictive value (NPV) of the faecal immunochemical test (FIT) at thresholds of 40 µg/g and 10 µg/g for advanced adenomas in participants who completed one, two or three tests and underwent colonic examination and did not have colorectal cancer diagnosed

FIT threshold (µg/g)	Test		Completed test*	Participants with advanced adenomas		Participants without advanced adenomas		Sensitivity (95% CI)	Specificity (95% CI)	PPV (95% CI)	NPV (95% CI)
			n	TP	FN	FP	TN	%	%	%	%
**40**		First	5196	75	365	234	4522	17.0 (13.6 to 20.9)	95.1 (94.4 to 95.7)	24.3 (19.6 to 29.4)	92.5 (91.8 to 93.3)
		Second†	4848	37	325	181	4305	10.2 (7.3 to 13.8)	96.0 (95.3 to 96.5)	17.0 (12.2 to 22.6)	93.0 (92.2 to 93.7)
		Third‡	4441	35	273	143	3990	11.4 (8.0 to 15.4)	96.5 (95.9 to 97.1)	19.7 (14.1 to 26.3)	93.6 (92.8 to 94.3)
	**Over two tests**	**CTA**§	5157	112	325	415	4305	25.6 (21.6 to 30.0)	91.2 (90.4 to 92.0)	21.3 (17.8 to 25.0)	93.0 (92.2 to 93.7)
		**PA**¶	5196	112	328	415	4341	25.5 (21.4 to 29.8)	91.3 (90.4 to 92.1)	21.3 (17.8 to 25.0)	93.0 (92.2 to 93.7)
	**Over three tests**	CTA**	4968	147	273	558	3990	35.0 (30.4 to 39.8)	87.7 (86.7 to 88.7)	20.9 (17.9 to 24.0)	93.6 (92.8 to 94.3)
		**PA**††	5196	147	293	558	4198	33.4 (29.0 to 38.0)	88.3 (87.3 to 89.2)	20.9 (17.9 to 24.0)	93.5 (92.7 to 94.2)
**10**		First	5196	145	295	573	4183	33.0 (28.6 to 37.6)	88.0 (87.0 to 88.9)	20.2 (17.3 to 23.3)	93.4 (92.6 to 94.1)
		Second†	4447	61	231	376	3779	20.9 (16.4 to 26.0)	91.0 (90.0 to 91.8)	14.0 (10.8 to 17.6)	94.2 (93.5 to 94.9)
		Third‡	3849	43	175	302	3329	19.7 (14.7 to 25.6)	91.7 (90.7 to 92.6)	12.5 (9.2 to 16.4)	95.0 (94.2 to 95.7)
	**Over two tests**	**CTA**§	5165	206	231	949	3779	47.1 (42.4 to 51.9)	79.9 (78.8 to 81.1)	17.8 (15.7 to 20.2)	94.2 (93.5 to 94.9)
		**PA**¶	5196	206	234	949	3807	46.8 (42.1 to 51.6)	80.0 (78.9 to 81.2)	17.8 (15.7 to 20.2)	94.2 (93.4 to 94.9)
	**Over three tests**	CTA**	5004	249	175	1251	3329	58.7 (53.9 to 63.5)	72.7 (71.4 to 74.0)	16.6 (14.8 to 18.6)	95.0 (94.2 to 95.7)
		**PA**††	5196	249	191	1251	3505	56.6 (51.8 to 61.3)	73.7 (72.4 to 74.9)	16.6 (14.8 to 18.6)	94.8 (94.1 to 95.5)

*Participants who tested positive at a given threshold at year 1 or 2 were excluded from subsequent analyses.

†Includes participants who completed their second FIT, either at year 2 or 3.

‡Includes participants who completed their third FIT.

§Includes participants who completed at least two FITs or who tested positive at year 1. Participants were classed as positive if they tested positive with either of their first two FITs.

¶Includes participants who completed at least one FIT. Participants were classed as positive if they tested positive with either of their first two FITs.

**Includes participants who completed all three FITs or who tested positive with any FIT. Participants were classed as positive if they tested positive with any FIT.

††Includes participants who completed at least one FIT. Participants were classed as positive if they tested positive with any FIT.

CTA, cumulative test analysis; FN, false negative; FP, false positive; PA, programme analysis; TN, true negative; TP, true positive

Although lacking in power, subgroup analyses revealed that sensitivities for AAs were slightly higher and specificities lower in men and older (>65 years) participants (online supplementary tables 7 and 8). Excluding participants with non-adenocarcinomas (squamous cell carcinoma: n=1; neuroendocrine tumours: n=2) saw 3-year programme sensitivities for CRC increase from 72.4% to 80.8% at 10 µg/g (data not shown). Excluding participants with colonic examinations of unknown quality (n=587) or with confirmed IBD (n=19) had negligible effects on diagnostic accuracy estimates, although excluding participants with confirmed or possible IBD (n=68) saw the 3-year programme sensitivity for CRC increase to 75.0% at 10 µg/g (data not shown).

The mean incremental cost per participant for 3 yearly colonoscopy surveillance versus 3 years of annual FIT (40 µg/g) was £365, and the total cost difference was £2 169 341. Incremental costs per additional AA and CRC detected by colonoscopy surveillance were £7354 and £180 778, respectively. Replacing colonoscopy with FIT surveillance nationally over a screening cycle would have a significant impact on budget savings: the total saving was estimated to be £4.7 million (online supplementary figure 1 and supplementary tables 9–12).

## Discussion

In this study, we evaluated the potential of annual FIT for postpolypectomy surveillance of intermediate-risk patients instead of 3 yearly colonoscopy. Annual FIT was well accepted with initial consent and FIT return of 74% and subsequent FIT return of 97% and less costly than colonoscopy surveillance. Sensitivities increased at lower FIT positivity thresholds; at a threshold of 10 µg/g, 3-year programme sensitivities were 72% for CRC and 57% for AAs.

Increased FIT sensitivity was accompanied by increased positivity and reduced specificity. Compared with one FIT at 40 µg/g, 3 years of low-threshold (10 µg/g) FIT was associated with five times as many positive and false positive results. Nevertheless, even with the latter regimen, cumulative positivity was only 29%. Substituting 3 yearly colonoscopy in intermediate-risk patients with low-threshold annual FIT could therefore reduce numbers of colonoscopies by 71%. However, this strategy would increase the risk of missing ACN; in our data, 28% of CRCs would have been missed. Although this percentage has limited precision owing to few CRC outcomes (n=29), it may cause concern as diagnostic delay can result in disease progression. Additional concern may arise from the fact that 30% of FIT-detected CRCs were not found until years 2 and 3, even with the low-threshold. If in practice FIT return following a negative result is depressed by false reassurance, the proportion of cancers missed by FIT could be even higher.

This discussion requires consideration of what is an ‘acceptable’ proportion of missed cancers, accounting for the possibility that missed cancers could be detected at treatable stages with continued FIT surveillance. This comes with the caveat that FIT return may fall following repeat negative testing. Delayed detection of AAs is less serious given the slower rate of transformation from adenoma to cancer,^1 37^ and a 3-year programme sensitivity of 57% may be considered acceptable.

It is interesting to consider these results in light of the findings of our ‘Intermediate Adenoma’ study.^8 38^ Analysing long-term CRC incidence in intermediate-risk patients postpolypectomy, we identified two risk subgroups. In the higher risk subgroup, comprising patients with suboptimal quality colonoscopies, adenomas ≥20 mm, high-grade adenomas or proximal polyps at baseline, colonoscopy surveillance was associated with a halving in incidence. In lower risk patients without these features, the value of surveillance was unclear. Therefore, while higher risk patients significantly benefit from 3 yearly colonoscopy, annual FIT may suffice for lower risk patients. FIT may also be useful in the surveillance of high-risk patients; for example, as a substitute for the 1-year colonoscopy. Future studies should investigate FIT performance in different risk subgroups.

Further research is needed to understand the influence of sex and age on FIT performance. Consistent with the literature, higher positivity was observed in men and older participants, reflecting the increased prevalence of ACN.^19 39 40^ Sensitivities were generally higher and specificities lower in men and older participants, although subgroup analyses lacked power. Other studies have reported variability in diagnostic accuracy of FIT by sex and age, leading to recommendations for individualisation of positivity thresholds.^19 39 40^ PPVs are particularly important as they indicate the probability of a colonoscopy being unnecessary following positive FIT. Here we did not detect a consistent pattern due to insufficient power, but a study of the Basque Country CRC Screening Programme found PPVs of FIT for ACN to be significantly higher in men than women at thresholds between 20 µg/g and 60 µg/g.^39^ Well-powered studies should investigate whether this is also the case in individuals undergoing surveillance.

FIT sensitivity varies according to lesion type and location. Here we reported higher sensitivities when the three non-adenocarcinomas were excluded; these were not detected by FIT, even at 10 µg/g. Poor sensitivity has also been reported for proximal and serrated lesions.^41^ Sensitivity may be improved by combining FIT with other faecal biomarker tests.^42^ A multitarget molecular stool test has shown greater sensitivity than FIT for ACN and for proximal and large serrated lesions, although has lower specificity.^41 43^ An evaluation of FIT and molecular stool testing as alternatives to colonoscopy surveillance is underway,^44^ although further research should examine how these perform in combination.

Intermediate-risk patients are currently recommended to undergo surveillance until they have two consecutive negative colonoscopies.^45^ Stopping rules for FIT-based surveillance would have to consider the susceptibility of some patients to repeatedly test false positive; something we observed in our analysis of BCSP participants who underwent gFOBT screening.^46^

To our knowledge, this study is the largest to have evaluated FIT for surveillance and the only to have evaluated FIT specifically in patients undergoing postpolypectomy surveillance. Previous studies included patients attending surveillance for personal or family histories of CRC.^22–27^ In four of these, sensitivities were calculated for a single FIT performed prior to surveillance colonoscopy.^23–26^ One study of 808 patients reported sensitivities of 70% for CRC and 44% for AAs for Hemeselect, a qualitative FIT that was deemed positive when the concentration of faecal haemoglobin exceeded 200–300 µg/g.^23^ The OC-Sensor (10 µg/g) achieved sensitivities for CRC and AAs, respectively, of 80% and 28% in a study of 1041 patients,^24^ and 100% and 44% in a study of 1000.^25^ A fourth study stratified sensitivity estimates by indication for surveillance, showing that in 348 patients undergoing surveillance for colorectal neoplasia, sensitivities of OC-Sensor (15 µg/g) were 100% for CRC and 68% for AAs.^26^

These studies reported higher sensitivities than we estimated for one FIT at 10 µg/g (52% for CRC; 33% for AAs), although small numbers of CRCs meant estimates were imprecise. Differences in patient mix, number of tests, scheduling of FIT relative to colonoscopy and the high quality of colonoscopy in England may have contributed to the discrepancy. There is one other study that evaluated FIT for surveillance with repeated testing^27^; among 1071 patients who completed at least one qualitative FIT between surveillance colonoscopies, sensitivities were 86% for CRC and 63% for AAs. In our study, sensitivities in patients completing at least one FIT was lower at 72% and 57% for CRC and AAs, respectively, over 3 years at 10 µg/g.

Strengths of our study include the relatively large sample of clearly defined patients undergoing postpolypectomy surveillance and evaluation of FIT at various thresholds and with multiple tests. We tested the robustness of our results in sensitivity analyses, observing little change when participants with unknown quality examinations and IBD were excluded. The high FIT return rates are a further strength, although it is likely that our inclusion criteria inflated rates at years 2 and 3 because only individuals consenting and returning an analysable year 1 FIT were offered further FITs, limiting the generalisability of our findings. This may have also biased our diagnostic accuracy estimates because individuals who provided consent but not an analysable FIT may have participated at subsequent years.

Limitations of this study include that it examined FIT performance only in relation to the first surveillance colonoscopy. We do not know the effect of continuing with FIT beyond 3 years. Furthermore, we assumed that colonic examinations detected all ACN; given that 98% of participants who underwent examination had colonoscopy, the ‘gold-standard’ investigation,^32^ this assumption is reasonable. That said, colonoscopy is not perfectly sensitive and occasionally misses adenomas and CRCs.^47 48^ A small percentage of participants received CT colonography only, which shows comparable sensitivity to colonoscopy for CRC and large polyps.^13^

Another limitation is that endoscopists may have been aware when examinations were for positive FIT results, which may have influenced the thoroughness of examination, potentially leading to inflated sensitivity estimates of FIT-positive colonoscopies. Additionally, we assumed that the population of ACN was static. Adenoma progression rates are thought to be low^1 37 49^; however, if neoplastic progression did occur, our reported sensitivities may be biased. The bias may be in either direction depending on the FIT threshold being considered, leading to both underestimation and overestimation of sensitivity. Biases may have resulted from missing information, as we did not know why some individuals had multiple baseline examinations and were therefore excluded or why some did not attend any colonic examination.

Interpretation of our results should take into account the specific nature of the study population, namely that it was drawn from BCSP participants assigned as intermediate-risk at colonoscopy performed following positive gFOBT. Generalisability is not limited by the use of a single FIT brand, as different brands can achieve almost equal sensitivity when thresholds are set to yield defined positivity rates.^33^

## Conclusion

Our results suggest that FIT could perform a role in postpolypectomy surveillance of intermediate-risk patients. If low-threshold annual FIT was implemented instead of 3 yearly colonoscopy, numbers of colonoscopies could be reduced by more than 70% with significant cost savings. However, this would come at the cost of missed ACN; depending on the threshold, annual FIT could miss 30%–40% of CRCs and 40%–70% of AAs. Further research is warranted before decisions are made about whether it is reasonable to adopt FIT for surveillance. Future studies should investigate FIT performance in longer term surveillance and within subgroups of intermediate-risk patients. Further economic analyses should examine the cost implications of CRCs and AAs missed by FIT and the impact on quality-adjusted life years.
