# Supplementary material for: Faecal immunochemical tests (FIT) versus colonoscopy for surveillance after screening and polypectomy: a diagnostic accuracy and cost-effectiveness study
Source: Gut. 2018 Dec 11;68(9):1642–52. doi: 10.1136/gutjnl-2018-317297 (PMC6709777; doi:10.1136/gutjnl-2018-317297)
Supplement: Supplementary file 1 [file gutjnl-2018-317297supp001.pdf]

SUPPLEMENTARY TABLES FOR THE MAIN DIAGNOSTIC ACCURACY STUDY

Supplementary Table 1. Uptake, positivity rate, and diagnostic yield of the faecal immunochemical test (FIT) at years one, two, and three and over all three years, stratified by sex

		Uptake			Positivity rate		Colonic exam performed				Diagnostic yield[e]			
		Invited	Completed FIT test[a]		Tested positive[b]		Any exam[c]		Colonoscopy[d]		Colorectal cancer		Advanced adenomas[f]	
**Sex**	**Year**	n	n	(%)	n	(%)	n	(%)	n	(%)	n	(%)	n	(%)
**Men**	1	5228	3892	(74.4)	257[g]	(6.6)	241	(93.8)	240	(99.6)	5	(2.1)	58	(24.1)
	2	3565	3469	(97.3)	169	(4.9)	156	(92.3)	152	(97.4)	3	(1.9)	28	(17.9)
	3	3346	3258	(97.4)	147	(4.5)	137	(93.2)	133	(97.1)	0	(0)	23	(16.8)
	Cumulative	5228	3892	(74.4)	573[h]	(14.7)	534	(93.2)	525	(98.3)	8	(1.5)	109	(20.4)
	**Routine year 3 colonic exam**						2931		2890	(98.6)	6	(0.2)	202	(6.9)
	**Entire study findings**						3465		3415	(98.6)	14	(0.4)	311	(9.0)
**Women**	1	2781	2046	(73.6)	89[g]	(4.3)	79	(88.8)	77	(97.5)	3	(3.8)	20	(25.3)
	2	1914	1860	(97.2)	67	(3.6)	60	(89.6)	60	(100)	4	(6.7)	9	(15.0)
	3	1833	1764	(96.2)	57	(3.2)	52	(91.2)	51	(98.1)	2	(3.8)	13	(25.0)
	Cumulative	2781	2046	(73.6)	213[h]	(10.4)	191	(89.7)	188	(98.4)	9	(4.7)	42	(22.0)
	**Routine year 3 colonic exam**						1569		1528	(97.4)	6	(0.4)	93	(5.9)
	**Entire study findings**						1760		1716	(97.5)	15	(0.9)	135	(7.7)

a. Participants who gave consent, returned an analysable FIT at year one, and did not subsequently withdraw from the study.
b. Percentages calculated using the number of participants who completed FIT as the denominator. In the pilot study, a threshold of 20µg haemoglobin (Hb)/g faeces was used to denote test positivity. The positivity threshold used in the rest of the study was 40µg Hb/g faeces.

c. Participants who underwent colonoscopy or computed tomography colonography. Percentages calculated using the number of FIT positive participants as the denominator.
d. Participants who had a colonoscopy. Percentages calculated using the number of participants who underwent colonic examination as the denominator.
e. Diagnostic yield in participants who underwent colonic examination.
f. Advanced adenomas were defined as adenomas ≥ 10mm, with villous or tubulovillous histology, or high-grade dysplasia.
g. Three participants tested positive at year one during the pilot study based on a threshold of 20µg Hb/g. They are included as FIT positive in this table even though their faecal haemoglobin levels were lower than the 40µg Hb/g threshold used in the rest of the study.
h. Participants who were FIT positive with any FIT, regardless of whether they had completed all FITs that they were offered.

Supplementary Table 2. Uptake, positivity rate, and diagnostic yield of the faecal immunochemical test (FIT) at years one, two, and three and over all three years, stratified by age at invitation date

		Uptake			Positivity rate		Colonic exam performed				Diagnostic yield[e]			
**Age at invitation date**		**Invited**	**Completed FIT test[a]**		**Tested positive[b]**		**Any exam[c]**		**Colonoscopy[d]**		**Colorectal cancer**		**Advanced adenomas[f]**	
	**Year**	n	n	(%)	n	(%)	n	(%)	n	(%)	n	(%)	n	(%)
**≤65 years**	1	3950	2877	(72.8)	147[g]	(5.1)	136	(92.5)	135	(99.3)	2	(1.5)	37	(27.2)
	2	2671	2595	(97.2)	105	(4.0)	98	(93.3)	95	(96.9)	3	(3.1)	14	(14.3)
	3	2548	2474	(97.1)	97	(3.9)	89	(91.8)	89	(100)	2	(2.2)	14	(15.7)
	Cumulative	3950	2877	(72.8)	349[h]	(12.1)	323	(92.6)	319	(98.8)	7	(2.2)	65	(20.1)
	**Routine year 3 colonic exam**						2228		2190	(98.3)	7	(0.3)	140	(6.3)
	**Entire study findings**						2551		2509	(98.4)	14	(0.5)	205	(8.0)
**>65 years**	1	4059	3061	(75.4)	199[g]	(6.5)	184	(92.5)	182	(98.9)	6	(3.3)	41	(22.3)
	2	2808	2734	(97.4)	131	(4.8)	118	(90.1)	117	(99.2)	4	(3.4)	23	(19.5)
	3	2631	2548	(96.8)	107	(4.2)	100	(93.5)	95	(95.0)	0	(0)	22	(22.0)
	Cumulative	4059	3061	(75.4)	437[h]	(14.3)	402	(92.0)	394	(98.0)	10	(2.5)	86	(21.4)
	**Routine year 3 colonic exam**						2272		2228	(98.1)	5	(0.2)	155	(6.8)
	**Entire study findings**						2674		2622	(98.1)	15	(0.6)	241	(9.0)

a. Participants who gave consent, returned an analysable FIT at year one, and did not subsequently withdraw from the study.
b. Percentages calculated using the number of participants who completed FIT as the denominator. In the pilot study, a threshold of 20μg haemoglobin (Hb)/g faeces was used to denote test positivity. The positivity threshold used in the rest of the study was 40μg Hb/g faeces.

c. Participants who underwent colonoscopy or computed tomography colonography. Percentages calculated using the number of FIT positive participants as the denominator.
d. Participants who had a colonoscopy. Percentages calculated using the number of participants who underwent colonic examination as the denominator.
e. Diagnostic yield in participants who underwent colonic examination.
f. Advanced adenomas were defined as adenomas ≥ 10mm, with villous or tubulovillous histology, or high-grade dysplasia.
g. Three participants tested positive at year one during the pilot study based on a threshold of 20µg Hb/g. They are included as FIT positive in this table even though their faecal haemoglobin levels were lower than the 40µg Hb/g threshold used in the rest of the study.
h. Participants who were FIT positive with any FIT, regardless of whether they had completed all FITs that they were offered.

Supplementary Table 3. Characteristics of the 29 participants diagnosed with colorectal cancer

Sex	Site of cancer	Stage of cancer	Type of cancer (ICD-02 histology code)		FIT result (µg/g)		
					First	Second	Third
M	Appendix	III	Mixed adenoneuroendocrine carcinoma	(8244)	61	*	*
M	Appendix	I	Carcinoid tumour	(8240)	2	†	†
M	Caecum	III	Adenocarcinoma	(8140)	0	0	†
F	Caecum	II	Adenocarcinoma	(8140)	786	*	*
F	Caecum	IV	Adenocarcinoma	(8140)	1	†	†
M	Caecum	II	Adenocarcinoma	(8140)	0	0	0
F	Ascending colon	III	Adenocarcinoma	(8140)	6	502	*
F	Ascending colon	I	Adenocarcinoma	(8140)	2	158	*
M	Ascending colon	III	Adenocarcinoma	(8140)	38	43	*
F	Ascending colon	II	Adenocarcinoma	(8140)	13	24	4
F	Ascending colon	III	Adenocarcinoma	(8140)	185	*	*
F	Transverse colon	I	Adenocarcinoma	(8140)	1	3	1
M	Transverse colon	II	Adenocarcinoma	(8140)	8	14	35
M	Transverse colon	I or II[§]	Adenocarcinoma	(8140)	0	113	*
F	Transverse colon	I	Mucinous adenocarcinoma	(8480)	2	9	52
M	Descending colon	I	Adenocarcinoma	(8140)	21	†	†
M	Sigmoid colon	Unknown	Adenocarcinoma	(8140)	271	*	*
M	Sigmoid colon	IV	Adenocarcinoma	(8140)	1937	*	*
F	Sigmoid colon	I	Adenocarcinoma in tubulovillous adenoma	(8263)	51	*	*
M	Sigmoid colon	II	Adenocarcinoma	(8140)	38	1523	*
F	Sigmoid colon	III	Adenocarcinoma (mucin secreting)	(8481)	5	13	5410
F	Rectosigmoid	III	Mucinous adenocarcinoma	(8480)	21	†	†
M	Rectosigmoid	I	Adenocarcinoma in adenomatous polyp	(8210)	97	*	*
M	Rectum	I	Squamous cell carcinoma	(8070)	3	†	†
F	Rectum	III	Adenocarcinoma	(8140)	11	95	*
F	Rectum	I	Adenocarcinoma in adenomatous polyp	(8210)	16	752	*
M	Rectum	I	Adenocarcinoma	(8140)	55	*	*
F	Rectum	Unknown	Carcinoid tumor	(8240)	0	1	‡
F	Rectum	Unknown	Adenocarcinoma	(8140)	5	0	0

M: male; F: female

*No FIT result as patient had tested positive with a previous FIT.

†No FIT result as patient had been diagnosed with cancer since testing negative with a previous FIT

‡No FIT result as patient did not complete FIT.

[§]Reports indicate no nodes or metastases but unclear whether the tumour is Stage I or II

Supplementary Table 4. Sensitivity, specificity, positive predictive value (PPV), and negative predictive value (NPV) of the faecal immunochemical test (FIT) at thresholds of 30µg/g and 20µg/g for colorectal cancer and advanced adenoma in participants who completed one, two, or three tests and underwent colonic examination

Outcome	FIT threshold (µg/g)	Test		Completed test[a]	Participants with colorectal cancer		Participants without colorectal cancer		Sensitivity (95% CI)	Specificity (95% CI)	PPV (95% CI)	NPV (95% CI)
				n	TP	FN	FP	TN	%	%	%	%
Colorectal cancer	30		1st	5225	10	19	366	4830	34.5 (17.9 - 54.3)	93.0 (92.2 - 93.6)	2.7 (1.3 - 4.8)	99.6 (99.4 - 99.8)
			2nd [b]	4806	5	9	264	4528	35.7 (12.8 - 64.9)	94.5 (93.8 - 95.1)	1.9 (0.6 - 4.3)	99.8 (99.6 - 99.9)
			3rd [c]	4350	3	4	216	4127	42.9 (9.9 - 81.6)	95.0 (94.3 - 95.7)	1.4 (0.3 - 4.0)	99.9 (99.8 - 100.0)
		Over 2 tests	CTA[d]	5182	15	9	630	4528	62.5 (40.6 - 81.2)	87.8 (86.9 - 88.7)	2.3 (1.3 - 3.8)	99.8 (99.6 - 99.9)
			PA[e]	5225	15	14	630	4566	51.7 (32.5 - 70.6)	87.9 (87.0 - 88.8)	2.3 (1.3 - 3.8)	99.7 (99.5 - 99.8)
		Over 3 tests	CTA[f]	4995	18	4	846	4127	81.8 (59.7 - 94.8)	83.0 (81.9 - 84.0)	2.1 (1.2 - 3.3)	99.9 (99.8 - 100.0)
			PA[g]	5225	18	11	846	4350	62.1 (42.3 - 79.3)	83.7 (82.7 - 84.7)	2.1 (1.2 - 3.3)	99.7 (99.5 - 99.9)
	20		1st	5225	12	17	469	4727	41.4 (23.5 - 61.1)	91.0 (90.2 - 91.7)	2.5 (1.3 - 4.3)	99.6 (99.4 - 99.8)
			2nd [b]	4708	6	8	323	4371	42.9 (17.7 - 71.1)	93.1 (92.4 - 93.8)	1.8 (0.7 - 3.9)	99.8 (99.6 - 99.9)
			3rd [c]	4201	3	3	263	3932	50.0 (11.8 - 88.2)	93.7 (93.0 - 94.4)	1.1 (0.2 - 3.3)	99.9 (99.8 - 100.0)
		Over 2 tests	CTA[d]	5189	18	8	792	4371	69.2 (48.2 - 85.7)	84.7 (83.6 - 85.6)	2.2 (1.3 - 3.5)	99.8 (99.6 - 99.9)
			PA[e]	5225	18	11	792	4404	62.1 (42.3 - 79.3)	84.8 (83.8 - 85.7)	2.2 (1.3 - 3.5)	99.8 (99.6 - 99.9)
		Over 3 tests	CTA[f]	5011	21	3	1055	3932	87.5 (67.6 - 97.3)	78.8 (77.7 - 80.0)	2.0 (1.2 - 3.0)	99.9 (99.8 - 100.0)
			PA[g]	5225	21	8	1055	4141	72.4 (52.8 - 87.3)	79.7 (78.6 - 80.8)	2.0 (1.2 - 3.0)	99.8 (99.6 - 99.9)
Advanced adenoma[h]	30		1st	5196	85	355	281	4475	19.3 (15.7 - 23.3)	94.1 (93.4 - 94.7)	23.2 (19.0 - 27.9)	92.7 (91.9 - 93.4)
			2nd [b]	4792	48	304	216	4224	13.6 (10.2 - 17.7)	95.1 (94.5 - 95.7)	18.2 (13.7 - 23.4)	93.3 (92.5 - 94.0)
			3rd [c]	4343	43	245	173	3882	14.9 (11.0 - 19.6)	95.7 (95.1 - 96.3)	19.9 (14.8 - 25.9)	94.1 (93.3 - 94.8)
		Over 2 tests	CTA[d]	5158	133	304	497	4224	30.4 (26.2 - 35.0)	89.5 (88.6 - 90.3)	21.1 (18.0 - 24.5)	93.3 (92.5 - 94.0)
			PA[e]	5196	133	307	497	4259	30.2 (26.0 - 34.8)	89.6 (88.6 - 90.4)	21.1 (18.0 - 24.5)	93.3 (92.5 - 94.0)
		Over 3 tests	CTA[f]	4973	176	245	670	3882	41.8 (37.0 - 46.7)	85.3 (84.2 - 86.3)	20.8 (18.1 - 23.7)	94.1 (93.3 - 94.8)
			PA[g]	5196	176	264	670	4086	40.0 (35.4 - 44.7)	85.9 (84.9 - 86.9)	20.8 (18.1 - 23.7)	93.9 (93.2 - 94.6)

	**20**		**1st**	5196	108	332	361	4395	24.5 (20.6 - 28.8)	92.4 (91.6 - 93.1)	23.0 (19.3 - 27.1)	93.0 (92.2 - 93.7)
			**2nd** [b]	4694	54	275	269	4096	16.4 (12.6 - 20.9)	93.8 (93.1 - 94.5)	16.7 (12.8 - 21.2)	93.7 (92.9 - 94.4)
			**3rd** [c]	4195	38	221	225	3711	14.7 (10.6 - 19.6)	94.3 (93.5 - 95.0)	14.4 (10.4 - 19.3)	94.4 (93.6 - 95.1)
		**Over 2 tests**	**CTA**[d]	5163	162	275	630	4096	37.1 (32.5 - 41.8)	86.7 (85.7 - 87.6)	20.5 (17.7 - 23.4)	93.7 (92.9 - 94.4)
			**PA**[e]	5196	162	278	630	4126	36.8 (32.3 - 41.5)	86.8 (85.8 - 87.7)	20.5 (17.7 - 23.4)	93.7 (92.9 - 94.4)
		**Over 3 tests**	**CTA**[f]	4987	200	221	855	3711	47.5 (42.6 - 52.4)	81.3 (80.1 - 82.4)	19.0 (16.6 - 21.5)	94.4 (93.6 - 95.1)
			**PA**[g]	5196	200	240	855	3901	45.5 (40.7 - 50.2)	82.0 (80.9 - 83.1)	19.0 (16.6 - 21.5)	94.2 (93.4 - 94.9)

PPV: positive predictive value; NPV: negative predictive value; CI: confidence interval; TP: true positive; FN: false negative; FP: false positive; FN: false negative; CTA: cumulative test analysis; PA: programme analysis.

a. Participants who tested positive at a given threshold at year one or two were excluded from subsequent analyses.
b. Includes participants who completed their second FIT, either at year two or three.
c. Includes participants who completed their third FIT.
d. Includes participants who completed at least two FITs or who tested positive at year one. Participants were classed as positive if they tested positive with either of their first two FITs.
e. Includes participants who completed at least one FIT. Participants were classed as positive if they tested positive with either of their first two FITs.
f. Includes participants who completed all three FITs or who tested positive with any FIT. Participants were classed as positive if they tested positive with any FIT.
g. Includes participants who completed at least one FIT. Participants were classed as positive if they tested positive with any FIT.
h. Excludes participants who had colorectal cancer diagnosed

Supplementary Table 5. Sensitivity, specificity, positive predictive value (PPV), and negative predictive value (NPV) of the faecal immunochemical test (FIT) for colorectal cancer at different thresholds in participants who completed one, two, or three tests and underwent colonic examination, stratified by sex

Sex	FIT threshold (µg/g)	Test		Completed test[a]	Participants with colorectal cancer		Participants without colorectal cancer		Sensitivity (95% CI)	Specificity (95% CI)	PPV (95% CI)	NPV (95% CI)
				n	TP	FN	FP	TN	%	%	%	%
Men	40		1st	3465	5	9	234	3217	35.7 (12.8 - 64.9)	93.2 (92.3 - 94.0)	2.1 (0.7 - 4.8)	99.7 (99.5 - 99.9)
			2nd[b]	3198	3	3	160	3032	50.0 (11.8 - 88.2)	95.0 (94.2 - 95.7)	1.8 (0.4 - 5.3)	99.9 (99.7 - 100.0)
			3rd[c]	2901	0	2	130	2769	0.0 (0.0 - 84.2)	95.5 (94.7 - 96.2)	0.0 (0.0 - 2.8)	99.9 (99.7 - 100.0)
		Over 2 tests	CTA[d]	3437	8	3	394	3032	72.7 (39.0 - 94.0)	88.5 (87.4 - 89.5)	2.0 (0.9 - 3.9)	99.9 (99.7 - 100.0)
			PA[e]	3465	8	6	394	3057	57.1 (28.9 - 82.3)	88.6 (87.5 - 89.6)	2.0 (0.9 - 3.9)	99.8 (99.6 - 99.9)
		Over 3 tests	CTA[f]	3303	8	2	524	2769	80.0 (44.4 - 97.5)	84.1 (82.8 - 85.3)	1.5 (0.7 - 2.9)	99.9 (99.7 - 100.0)
			PA[g]	3465	8	6	524	2927	57.1 (28.9 - 82.3)	84.8 (83.6 - 86.0)	1.5 (0.7 - 2.9)	99.8 (99.6 - 99.9)
	30		1st	3465	7	7	277	3174	50.0 (23.0 - 77.0)	92.0 (91.0 - 92.9)	2.5 (1.0 - 5.0)	99.8 (99.5 - 99.9)
			2nd[b]	3154	1	3	197	2953	25.0 (0.6 - 80.6)	93.7 (92.8 - 94.6)	0.5 (0.0 - 2.8)	99.9 (99.7 - 100.0)
			3rd[c]	2825	1	1	155	2668	50.0 (1.3 - 98.7)	94.5 (93.6 - 95.3)	0.6 (0.0 - 3.5)	100 (99.8 - 100.0)
		Over 2 tests	CTA[d]	3438	8	3	474	2953	72.7 (39.0 - 94.0)	86.2 (85.0 - 87.3)	1.7 (0.7 - 3.2)	99.9 (99.7 - 100.0)
			PA[e]	3465	8	6	474	2977	57.1 (28.9 - 82.3)	86.3 (85.1 - 87.4)	1.7 (0.7 - 3.2)	99.8 (99.6 - 99.9)
		Over 3 tests	CTA[f]	3307	9	1	629	2668	90.0 (55.5 - 99.7)	80.9 (79.5 - 82.3)	1.4 (0.6 - 2.7)	100 (99.8 - 100.0)
			PA[g]	3465	9	5	629	2822	64.3 (35.1 - 87.2)	81.8 (80.4 - 83.0)	1.4 (0.6 - 2.7)	99.8 (99.6 - 99.9)
	20		1st	3465	8	6	348	3103	57.1 (28.9 - 82.3)	89.9 (88.9 - 90.9)	2.2 (1.0 - 4.4)	99.8 (99.6 - 99.9)
			2nd[b]	3085	1	3	234	2847	25.0 (0.6 - 80.6)	92.4 (91.4 - 93.3)	0.4 (0.0 - 2.3)	99.9 (99.7 - 100.0)
			3rd[c]	2724	1	1	194	2528	50.0 (1.3 - 98.7)	92.9 (91.8 - 93.8)	0.5 (0.0 - 2.8)	100 (99.8 - 100.0)
		Over 2 tests	CTA[d]	3441	9	3	582	2847	75.0 (42.8 - 94.5)	83.0 (81.7 - 84.3)	1.5 (0.7 - 2.9)	99.9 (99.7 - 100.0)
			PA[e]	3465	9	5	582	2869	64.3 (35.1 - 87.2)	83.1 (81.8 - 84.4)	1.5 (0.7 - 2.9)	99.8 (99.6 - 99.9)
		Over 3 tests	CTA[f]	3315	10	1	776	2528	90.9 (58.7 - 99.8)	76.5 (75.0 - 77.9)	1.3 (0.6 - 2.3)	100 (99.8 - 100.0)
			PA[g]	3465	10	4	776	2675	71.4 (41.9 - 91.6)	77.5 (76.1 - 78.9)	1.3 (0.6 - 2.3)	99.9 (99.6 - 100.0)

	**10**		**1st**	3465	8	6	518	2933	57.1 (28.9 - 82.3)	85.0 (83.8 - 86.2)	1.5 (0.7 - 3.0)	99.8 (99.6 - 99.9)
			**2nd** [b]	2916	2	2	309	2603	50.0 (6.8 - 93.2)	89.4 (88.2 - 90.5)	0.6 (0.1 - 2.3)	99.9 (99.7 - 100.0)
			**3rd** [c]	2488	0	1	245	2242	0.0 (0.0 - 97.5)	90.1 (88.9 - 91.3)	0.0 (0.0 - 1.5)	100 (99.8 - 100.0)
		**Over 2 tests**	**CTA** [d]	3442	10	2	827	2603	83.3 (51.6 - 97.9)	75.9 (74.4 - 77.3)	1.2 (0.6 - 2.2)	99.9 (99.7 - 100.0)
			**PA** [e]	3465	10	4	827	2624	71.4 (41.9 - 91.6)	76.0 (74.6 - 77.5)	1.2 (0.6 - 2.2)	99.8 (99.6 - 100.0)
		**Over 3 tests**	**CTA** [f]	3325	10	1	1072	2242	90.9 (58.7 - 99.8)	67.7 (66.0 - 69.2)	0.9 (0.4 - 1.7)	100 (99.8 - 100.0)
			**PA** [g]	3465	10	4	1072	2379	71.4 (41.9 - 91.6)	68.9 (67.4 - 70.5)	0.9 (0.4 - 1.7)	99.8 (99.6 - 100.0)
**Women**	**40**		**1st**	1760	3	12	75	1670	20.0 (4.3 - 48.1)	95.7 (94.6 - 96.6)	3.8 (0.8 - 10.8)	99.3 (98.8 - 99.6)
			**2nd** [b]	1666	4	6	58	1598	40.0 (12.2 - 73.8)	96.5 (95.5 - 97.3)	6.5 (1.8 - 15.7)	99.6 (99.2 - 99.9)
			**3rd** [c]	1547	2	3	48	1494	40.0 (5.3 - 85.3)	96.9 (95.9 - 97.7)	4.0 (0.5 - 13.7)	99.8 (99.4 - 100.0)
		**Over 2 tests**	**CTA** [d]	1744	7	6	133	1598	53.8 (25.1 - 80.8)	92.3 (91.0 - 93.5)	5.0 (2.0 - 10.0)	99.6 (99.2 - 99.9)
			**PA** [e]	1760	7	8	133	1612	46.7 (21.3 - 73.4)	92.4 (91.0 - 93.6)	5.0 (2.0 - 10.0)	99.5 (99.0 - 99.8)
		**Over 3 tests**	**CTA** [f]	1687	9	3	181	1494	75.0 (42.8 - 94.5)	89.2 (87.6 - 90.6)	4.7 (2.2 - 8.8)	99.8 (99.4 - 100.0)
			**PA** [g]	1760	9	6	181	1564	60.0 (32.3 - 83.7)	89.6 (88.1 - 91.0)	4.7 (2.2 - 8.8)	99.6 (99.2 - 99.9)
	**30**		**1st**	1760	3	12	89	1656	20.0 (4.3 - 48.1)	94.9 (93.8 - 95.9)	3.3 (0.7 - 9.2)	99.3 (98.7 - 99.6)
			**2nd** [b]	1652	4	6	67	1575	40.0 (12.2 - 73.8)	95.9 (94.8 - 96.8)	5.6 (1.6 - 13.8)	99.6 (99.2 - 99.9)
			**3rd** [c]	1525	2	3	61	1459	40.0 (5.3 - 85.3)	96.0 (94.9 - 96.9)	3.2 (0.4 - 11.0)	99.8 (99.4 - 100.0)
		**Over 2 tests**	**CTA** [d]	1744	7	6	156	1575	53.8 (25.1 - 80.8)	91.0 (89.5 - 92.3)	4.3 (1.7 - 8.6)	99.6 (99.2 - 99.9)
			**PA** [e]	1760	7	8	156	1589	46.7 (21.3 - 73.4)	91.1 (89.6 - 92.4)	4.3 (1.7 - 8.6)	99.5 (99.0 - 99.8)
		**Over 3 tests**	**CTA** [f]	1688	9	3	217	1459	75.0 (42.8 - 94.5)	87.1 (85.4 - 88.6)	4.0 (1.8 - 7.4)	99.8 (99.4 - 100.0)
			**PA** [g]	1760	9	6	217	1528	60.0 (32.3 - 83.7)	87.6 (85.9 - 89.1)	4.0 (1.8 - 7.4)	99.6 (99.2 - 99.9)
	**20**		**1st**	1760	4	11	121	1624	26.7 (7.8 - 55.1)	93.1 (91.8 - 94.2)	3.2 (0.9 - 8.0)	99.3 (98.8 - 99.7)
			**2nd** [b]	1623	5	5	89	1524	50.0 (18.7 - 81.3)	94.5 (93.3 - 95.5)	5.3 (1.7 - 12.0)	99.7 (99.2 - 99.9)
			**3rd** [c]	1477	2	2	69	1404	50.0 (6.8 - 93.2)	95.3 (94.1 - 96.3)	2.8 (0.3 - 9.8)	99.9 (99.5 - 100.0)
		**Over 2 tests**	**CTA** [d]	1748	9	5	210	1524	64.3 (35.1 - 87.2)	87.9 (86.3 - 89.4)	4.1 (1.9 - 7.7)	99.7 (99.2 - 99.9)
			**PA** [e]	1760	9	6	210	1535	60.0 (32.3 - 83.7)	88.0 (86.3 - 89.5)	4.1 (1.9 - 7.7)	99.6 (99.2 - 99.9)
		**Over 3 tests**	**CTA** [f]	1696	11	2	279	1404	84.6 (54.6 - 98.1)	83.4 (81.6 - 85.2)	3.8 (1.9 - 6.7)	99.9 (99.5 - 100.0)
			**PA** [g]	1760	11	4	279	1466	73.3 (44.9 - 92.2)	84.0 (82.2 - 85.7)	3.8 (1.9 - 6.7)	99.7 (99.3 - 99.9)
	**10**		**1st**	1760	7	8	200	1545	46.7 (21.3 - 73.4)	88.5 (87.0 - 90.0)	3.4 (1.4 - 6.8)	99.5 (99.0 - 99.8)

		**2nd**[b]	1542	3	4	128	1407	42.9 (9.9 - 81.6)	91.7 (90.2 - 93.0)	2.3 (0.5 - 6.5)	99.7 (99.3 - 99.9)
		**3rd**[c]	1365	1	2	100	1262	33.3 (0.8 - 90.6)	92.7 (91.1 - 94.0)	1.0 (0.0 - 5.4)	99.8 (99.4 - 100.0)
		**Over 2 tests** **CTA**[d]	1749	10	4	328	1407	71.4 (41.9 - 91.6)	81.1 (79.2 - 82.9)	3.0 (1.4 - 5.4)	99.7 (99.3 - 99.9)
		**PA**[e]	1760	10	5	328	1417	66.7 (38.4 - 88.2)	81.2 (79.3 - 83.0)	3.0 (1.4 - 5.4)	99.6 (99.2 - 99.9)
		**Over 3 tests** **CTA**[f]	1703	11	2	428	1262	84.6 (54.6 - 98.1)	74.7 (72.5 - 76.7)	2.5 (1.3 - 4.4)	99.8 (99.4 - 100.0)
		**PA**[g]	1760	11	4	428	1317	73.3 (44.9 - 92.2)	75.5 (73.4 - 77.5)	2.5 (1.3 - 4.4)	99.7 (99.2 - 99.9)

PPV: positive predictive value; NPV: negative predictive value; CI: confidence interval; TP: true positive; FN: false negative; FP: false positive; FN: false negative; CTA: cumulative test analysis; PA: programme analysis.

a. Participants who tested positive at a given threshold at year one or two were excluded from subsequent analyses.
b. Includes participants who completed their second FIT, either at year two or three.
c. Includes participants who completed their third FIT.
d. Includes participants who completed at least two FITs or who tested positive at year one. Participants were classed as positive if they tested positive with either of their first two FITs.
e. Includes participants who completed at least one FIT. Participants were classed as positive if they tested positive with either of their first two FITs.
f. Includes participants who completed all three FITs or who tested positive with any FIT. Participants were classed as positive if they tested positive with any FIT.
g. Includes participants who completed at least one FIT. Participants were classed as positive if they tested positive with any FIT.

Supplementary Table 6. Sensitivity, specificity, positive predictive value (PPV), and negative predictive value (NPV) of the faecal immunochemical test (FIT) for colorectal cancer at different thresholds in participants who completed one, two, or three tests and underwent colonic examination, stratified by age at invitation date

Age at invitation date	FIT threshold (µg/g)	Test		Completed test[a]	Participants with colorectal cancer		Participants without colorectal cancer		Sensitivity (95% CI)	Specificity (95% CI)	PPV (95% CI)	NPV (95% CI)
				n	TP	FN	FP	TN	%	%	%	%
≤65 years	40		1st	2551	2	12	132	2405	14.3 (1.8 - 42.8)	94.8 (93.9 - 95.6)	1.5 (0.2 - 5.3)	99.5 (99.1 - 99.7)
			2nd[b]	2393	3	6	101	2283	33.3 (7.5 - 70.1)	95.8 (94.9 - 96.5)	2.9 (0.6 - 8.2)	99.7 (99.4 - 99.9)
			3rd[c]	2187	2	2	81	2102	50.0 (6.8 - 93.2)	96.3 (95.4 - 97.0)	2.4 (0.3 - 8.4)	99.9 (99.7 - 100.0)
		Over 2 tests	CTA[d]	2527	5	6	233	2283	45.5 (16.7 - 76.6)	90.7 (89.5 - 91.8)	2.1 (0.7 - 4.8)	99.7 (99.4 - 99.9)
			PA[e]	2551	5	9	233	2304	35.7 (12.8 - 64.9)	90.8 (89.6 - 91.9)	2.1 (0.7 - 4.8)	99.6 (99.3 - 99.8)
		Over 3 tests	CTA[f]	2425	7	2	314	2102	77.8 (40.0 - 97.2)	87.0 (85.6 - 88.3)	2.2 (0.9 - 4.4)	99.9 (99.7 - 100.0)
			PA[g]	2551	7	7	314	2223	50.0 (23.0 - 77.0)	87.6 (86.3 - 88.9)	2.2 (0.9 - 4.4)	99.7 (99.4 - 99.9)
	30		1st	2551	3	11	154	2383	21.4 (4.7 - 50.8)	93.9 (92.9 - 94.8)	1.9 (0.4 - 5.5)	99.5 (99.2 - 99.8)
			2nd[b]	2371	2	6	114	2249	25.0 (3.2 - 65.1)	95.2 (94.2 - 96.0)	1.7 (0.2 - 6.1)	99.7 (99.4 - 99.9)
			3rd[c]	2154	3	1	102	2048	75.0 (19.4 - 99.4)	95.3 (94.3 - 96.1)	2.9 (0.6 - 8.1)	100 (99.7 - 100.0)
		Over 2 tests	CTA[d]	2528	5	6	268	2249	45.5 (16.7 - 76.6)	89.4 (88.1 - 90.5)	1.8 (0.6 - 4.2)	99.7 (99.4 - 99.9)
			PA[e]	2551	5	9	268	2269	35.7 (12.8 - 64.9)	89.4 (88.2 - 90.6)	1.8 (0.6 - 4.2)	99.6 (99.3 - 99.8)
		Over 3 tests	CTA[f]	2427	8	1	370	2048	88.9 (51.8 - 99.7)	84.7 (83.2 - 86.1)	2.1 (0.9 - 4.1)	100 (99.7 - 100.0)
			PA[g]	2551	8	6	370	2167	57.1 (28.9 - 82.3)	85.4 (84.0 - 86.8)	2.1 (0.9 - 4.1)	99.7 (99.4 - 99.9)
	20		1st	2551	3	11	199	2338	21.4 (4.7 - 50.8)	92.2 (91.0 - 93.2)	1.5 (0.3 - 4.3)	99.5 (99.2 - 99.8)
			2nd[b]	2329	2	6	151	2170	25.0 (3.2 - 65.1)	93.5 (92.4 - 94.5)	1.3 (0.2 - 4.6)	99.7 (99.4 - 99.9)
			3rd[c]	2082	3	1	125	1953	75.0 (19.4 - 99.4)	94.0 (92.9 - 95.0)	2.3 (0.5 - 6.7)	99.9 (99.7 - 100.0)
		Over 2 tests	CTA[d]	2531	5	6	350	2170	45.5 (16.7 - 76.6)	86.1 (84.7 - 87.4)	1.4 (0.5 - 3.3)	99.7 (99.4 - 99.9)
			PA[e]	2551	5	9	350	2187	35.7 (12.8 - 64.9)	86.2 (84.8 - 87.5)	1.4 (0.5 - 3.3)	99.6 (99.2 - 99.8)
		Over 3 tests	CTA[f]	2437	8	1	475	1953	88.9 (51.8 - 99.7)	80.4 (78.8 - 82.0)	1.7 (0.7 - 3.2)	99.9 (99.7 - 100.0)
			PA[g]	2551	8	6	475	2062	57.1 (28.9 - 82.3)	81.3 (79.7 - 82.8)	1.7 (0.7 - 3.2)	99.7 (99.4 - 99.9)

	**10**		**1st**	2551	4	10	313	2224	28.6 (8.4 - 58.1)	87.7 (86.3 - 88.9)	1.3 (0.3 - 3.2)	99.6 (99.2 - 99.8)
			**2nd** [b]	2216	3	4	210	1999	42.9 (9.9 - 81.6)	90.5 (89.2 - 91.7)	1.4 (0.3 - 4.1)	99.8 (99.5 - 99.9)
			**3rd** [c]	1918	1	1	175	1741	50.0 (1.3 - 98.7)	90.9 (89.5 - 92.1)	0.6 (0.0 - 3.1)	99.9 (99.7 - 100.0)
		**Over 2 tests**	**CTA** [d]	2533	7	4	523	1999	63.6 (30.8 - 89.1)	79.3 (77.6 - 80.8)	1.3 (0.5 - 2.7)	99.8 (99.5 - 99.9)
			**PA** [e]	2551	7	7	523	2014	50.0 (23.0 - 77.0)	79.4 (77.8 - 80.9)	1.3 (0.5 - 2.7)	99.7 (99.3 - 99.9)
		**Over 3 tests**	**CTA** [f]	2448	8	1	698	1741	88.9 (51.8 - 99.7)	71.4 (69.5 - 73.2)	1.1 (0.5 - 2.2)	99.9 (99.7 - 100.0)
			**PA** [g]	2551	8	6	698	1839	57.1 (28.9 - 82.3)	72.5 (70.7 - 74.2)	1.1 (0.5 - 2.2)	99.7 (99.3 - 99.9)
**>65 years**	**40**		**1st**	2674	6	9	177	2482	40.0 (16.3 - 67.7)	93.3 (92.3 - 94.3)	3.3 (1.2 - 7.0)	99.6 (99.3 - 99.8)
			**2nd** [b]	2471	4	3	117	2347	57.1 (18.4 - 90.1)	95.3 (94.3 - 96.1)	3.3 (0.9 - 8.2)	99.9 (99.6 - 100.0)
			**3rd** [c]	2261	0	3	97	2161	0.0 (0.0 - 70.8)	95.7 (94.8 - 96.5)	0.0 (0.0 - 3.7)	99.9 (99.6 - 100.0)
		**Over 2 tests**	**CTA** [d]	2654	10	3	294	2347	76.9 (46.2 - 95.0)	88.9 (87.6 - 90.0)	3.3 (1.6 - 6.0)	99.9 (99.6 - 100.0)
			**PA** [e]	2674	10	5	294	2365	66.7 (38.4 - 88.2)	88.9 (87.7 - 90.1)	3.3 (1.6 - 6.0)	99.8 (99.5 - 99.9)
		**Over 3 tests**	**CTA** [f]	2565	10	3	391	2161	76.9 (46.2 - 95.0)	84.7 (83.2 - 86.1)	2.5 (1.2 - 4.5)	99.9 (99.6 - 100.0)
			**PA** [g]	2674	10	5	391	2268	66.7 (38.4 - 88.2)	85.3 (83.9 - 86.6)	2.5 (1.2 - 4.5)	99.8 (99.5 - 99.9)
	**30**		**1st**	2674	7	8	212	2447	46.7 (21.3 - 73.4)	92.0 (90.9 - 93.0)	3.2 (1.3 - 6.5)	99.7 (99.4 - 99.9)
			**2nd** [b]	2435	3	3	150	2279	50.0 (11.8 - 88.2)	93.8 (92.8 - 94.7)	2.0 (0.4 - 5.6)	99.9 (99.6 - 100.0)
			**3rd** [c]	2196	0	3	114	2079	0.0 (0.0 - 70.8)	94.8 (93.8 - 95.7)	0.0 (0.0 - 3.2)	99.9 (99.6 - 100.0)
		**Over 2 tests**	**CTA** [d]	2654	10	3	362	2279	76.9 (46.2 - 95.0)	86.3 (84.9 - 87.6)	2.7 (1.3 - 4.9)	99.9 (99.6 - 100.0)
			**PA** [e]	2674	10	5	362	2297	66.7 (38.4 - 88.2)	86.4 (85.0 - 87.7)	2.7 (1.3 - 4.9)	99.8 (99.5 - 99.9)
		**Over 3 tests**	**CTA** [f]	2568	10	3	476	2079	76.9 (46.2 - 95.0)	81.4 (79.8 - 82.9)	2.1 (1.0 - 3.8)	99.9 (99.6 - 100.0)
			**PA** [g]	2674	10	5	476	2183	66.7 (38.4 - 88.2)	82.1 (80.6 - 83.5)	2.1 (1.0 - 3.8)	99.8 (99.5 - 99.9)
	**20**		**1st**	2674	9	6	270	2389	60.0 (32.3 - 83.7)	89.8 (88.6 - 91.0)	3.2 (1.5 - 6.0)	99.7 (99.5 - 99.9)
			**2nd** [b]	2379	4	2	172	2201	66.7 (22.3 - 95.7)	92.8 (91.6 - 93.8)	2.3 (0.6 - 5.7)	99.9 (99.7 - 100.0)
			**3rd** [c]	2119	0	2	138	1979	0.0 (0.0 - 84.2)	93.5 (92.3 - 94.5)	0.0 (0.0 - 2.6)	99.9 (99.6 - 100.0)
		**Over 2 tests**	**CTA** [d]	2658	13	2	442	2201	86.7 (59.5 - 98.3)	83.3 (81.8 - 84.7)	2.9 (1.5 - 4.8)	99.9 (99.7 - 100.0)
			**PA** [e]	2674	13	2	442	2217	86.7 (59.5 - 98.3)	83.4 (81.9 - 84.8)	2.9 (1.5 - 4.8)	99.9 (99.7 - 100.0)
		**Over 3 tests**	**CTA** [f]	2574	13	2	580	1979	86.7 (59.5 - 98.3)	77.3 (75.7 - 78.9)	2.2 (1.2 - 3.7)	99.9 (99.6 - 100.0)
			**PA** [g]	2674	13	2	580	2079	86.7 (59.5 - 98.3)	78.2 (76.6 - 79.7)	2.2 (1.2 - 3.7)	99.9 (99.7 - 100.0)
	**10**		**1st**	2674	11	4	405	2254	73.3 (44.9 - 92.2)	84.8 (83.3 - 86.1)	2.6 (1.3 - 4.7)	99.8 (99.5 - 100.0)

			**2nd**[b]	2242	2	2	227	2011	50.0 (6.8 - 93.2)	89.9 (88.5 - 91.1)	0.9 (0.1 - 3.1)	99.9 (99.6 - 100.0)
			**3rd**[c]	1935	0	2	170	1763	0.0 (0.0 - 84.2)	91.2 (89.9 - 92.4)	0.0 (0.0 - 2.1)	99.9 (99.6 - 100.0)
		**Over 2 tests**	**CTA**[d]	2658	13	2	632	2011	86.7 (59.5 - 98.3)	76.1 (74.4 - 77.7)	2.0 (1.1 - 3.4)	99.9 (99.6 - 100.0)
			**PA**[e]	2674	13	2	632	2027	86.7 (59.5 - 98.3)	76.2 (74.6 - 77.8)	2.0 (1.1 - 3.4)	99.9 (99.6 - 100.0)
		**Over 3 tests**	**CTA**[f]	2580	13	2	802	1763	86.7 (59.5 - 98.3)	68.7 (66.9 - 70.5)	1.6 (0.9 - 2.7)	99.9 (99.6 - 100.0)
			**PA**[g]	2674	13	2	802	1857	86.7 (59.5 - 98.3)	69.8 (68.1 - 71.6)	1.6 (0.9 - 2.7)	99.9 (99.6 - 100.0)

PPV: positive predictive value; NPV: negative predictive value; CI: confidence interval; TP: true positive; FN: false negative; FP: false positive; FN: false negative; CTA: cumulative test analysis; PA: programme analysis.

a. Participants who tested positive at a given threshold at year one or two were excluded from subsequent analyses.
b. Includes participants who completed their second FIT, either at year two or three.
c. Includes participants who completed their third FIT.
d. Includes participants who completed at least two FITs or who tested positive at year one. Participants were classed as positive if they tested positive with either of their first two FITs.
e. Includes participants who completed at least one FIT. Participants were classed as positive if they tested positive with either of their first two FITs.
f. Includes participants who completed all three FITs or who tested positive with any FIT. Participants were classed as positive if they tested positive with any FIT
g. Includes participants who completed at least one FIT. Participants were classed as positive if they tested positive with any FIT.

Supplementary Table 7. Sensitivity, specificity, positive predictive value (PPV), and negative predictive value (NPV) of the faecal immunochemical test (FIT) for advanced adenomas at different thresholds in participants who completed one, two, or three tests and underwent colonic examination and did not have colorectal cancer diagnosed, stratified by sex

Sex	FIT threshold (µg/g)	Test		Completed test[a]	Participants with advanced adenomas[b]		Participants without advanced adenomas[b]		Sensitivity (95% CI)	Specificity (95% CI)	PPV (95% CI)	NPV (95% CI)
				n	TP	FN	FP	TN	%	%	%	%
**Men**	**40**		**1st**	3451	57	251	177	2966	18.5 (14.3 - 23.3)	94.4 (93.5 - 95.1)	24.4 (19.0 - 30.4)	92.2 (91.2 - 93.1)
			**2nd** [c]	3192	29	221	131	2811	11.6 (7.9 - 16.2)	95.5 (94.7 - 96.3)	18.1 (12.5 - 25.0)	92.7 (91.7 - 93.6)
			**3rd** [d]	2899	22	186	108	2583	10.6 (6.7 - 15.6)	96.0 (95.2 - 96.7)	16.9 (10.9 - 24.5)	93.3 (92.3 - 94.2)
		**Over 2 tests**	**CTA**[e]	3426	86	221	308	2811	28.0 (23.1 - 33.4)	90.1 (89.0 - 91.2)	21.8 (17.8 - 26.2)	92.7 (91.7 - 93.6)
			**PA**[f]	3451	86	222	308	2835	27.9 (23.0 - 33.3)	90.2 (89.1 - 91.2)	21.8 (17.8 - 26.2)	92.7 (91.8 - 93.6)
		**Over 3 tests**	**CTA**[g]	3293	108	186	416	2583	36.7 (31.2 - 42.5)	86.1 (84.8 - 87.3)	20.6 (17.2 - 24.3)	93.3 (92.3 - 94.2)
			**PA**[h]	3451	108	200	416	2727	35.1 (29.7 - 40.7)	86.8 (85.5 - 87.9)	20.6 (17.2 - 24.3)	93.2 (92.2 - 94.1)
	**30**		**1st**	3451	65	243	212	2931	21.1 (16.7 - 26.1)	93.3 (92.3 - 94.1)	23.5 (18.6 - 28.9)	92.3 (91.4 - 93.2)
			**2nd** [c]	3150	37	205	160	2748	15.3 (11.0 - 20.5)	94.5 (93.6 - 95.3)	18.8 (13.6 - 24.9)	93.1 (92.1 - 93.9)
			**3rd** [d]	2823	28	164	127	2504	14.6 (9.9 - 20.4)	95.2 (94.3 - 96.0)	18.1 (12.4 - 25.0)	93.9 (92.9 - 94.7)
		**Over 2 tests**	**CTA**[e]	3427	102	205	372	2748	33.2 (28.0 - 38.8)	88.1 (86.9 - 89.2)	21.5 (17.9 - 25.5)	93.1 (92.1 - 93.9)
			**PA**[f]	3451	102	206	372	2771	33.1 (27.9 - 38.7)	88.2 (87.0 - 89.3)	21.5 (17.9 - 25.5)	93.1 (92.1 - 94.0)
		**Over 3 tests**	**CTA**[g]	3297	130	164	499	2504	44.2 (38.5 - 50.1)	83.4 (82.0 - 84.7)	20.7 (17.6 - 24.0)	93.9 (92.9 - 94.7)
			**PA**[h]	3451	130	178	499	2644	42.2 (36.6 - 47.9)	84.1 (82.8 - 85.4)	20.7 (17.6 - 24.0)	93.7 (92.7 - 94.6)
	**20**		**1st**	3451	83	225	265	2878	26.9 (22.1 - 32.3)	91.6 (90.5 - 92.5)	23.9 (19.5 - 28.7)	92.7 (91.8 - 93.6)
			**2nd** [c]	3081	41	183	193	2664	18.3 (13.5 - 24.0)	93.2 (92.3 - 94.1)	17.5 (12.9 - 23.0)	93.6 (92.6 - 94.4)
			**3rd** [d]	2722	23	147	171	2381	13.5 (8.8 - 19.6)	93.3 (92.3 - 94.2)	11.9 (7.7 - 17.3)	94.2 (93.2 - 95.1)
		**Over 2 tests**	**CTA**[e]	3429	124	183	458	2664	40.4 (34.9 - 46.1)	85.3 (84.0 - 86.6)	21.3 (18.0 - 24.9)	93.6 (92.6 - 94.4)
			**PA**[f]	3451	124	184	458	2685	40.3 (34.7 - 46.0)	85.4 (84.1 - 86.6)	21.3 (18.0 - 24.9)	93.6 (92.6 - 94.5)
		**Over 3 tests**	**CTA**[g]	3304	147	147	629	2381	50.0 (44.1 - 55.9)	79.1 (77.6 - 80.5)	18.9 (16.2 - 21.9)	94.2 (93.2 - 95.1)
			**PA**[h]	3451	147	161	629	2514	47.7 (42.0 - 53.5)	80.0 (78.5 - 81.4)	18.9 (16.2 - 21.9)	94.0 (93.0 - 94.9)

	**10**		**1st**	3451	108	200	410	2733	35.1 (29.7 - 40.7)	87.0 (85.7 - 88.1)	20.8 (17.4 - 24.6)	93.2 (92.2 - 94.1)
			**2nd**[c]	2912	44	155	265	2448	22.1 (16.5 - 28.5)	90.2 (89.1 - 91.3)	14.2 (10.5 - 18.6)	94.0 (93.1 - 94.9)
			**3rd**[d]	2487	28	117	217	2125	19.3 (13.2 - 26.7)	90.7 (89.5 - 91.9)	11.4 (7.7 - 16.1)	94.8 (93.8 - 95.7)
		**Over 2 tests**	**CTA**[e]	3430	152	155	675	2448	49.5 (43.8 - 55.2)	78.4 (76.9 - 79.8)	18.4 (15.8 - 21.2)	94.0 (93.1 - 94.9)
			**PA**[f]	3451	152	156	675	2468	49.4 (43.6 - 55.1)	78.5 (77.0 - 79.9)	18.4 (15.8 - 21.2)	94.1 (93.1 - 94.9)
		**Over 3 tests**	**CTA**[g]	3314	180	117	892	2125	60.6 (54.8 - 66.2)	70.4 (68.8 - 72.1)	16.8 (14.6 - 19.2)	94.8 (93.8 - 95.7)
			**PA**[h]	3451	180	128	892	2251	58.4 (52.7 - 64.0)	71.6 (70.0 - 73.2)	16.8 (14.6 - 19.2)	94.6 (93.6 - 95.5)
**Women**	**40**		**1st**	1745	18	114	57	1556	13.6 (8.3 - 20.7)	96.5 (95.4 - 97.3)	24.0 (14.9 - 35.3)	93.2 (91.9 - 94.3)
			**2nd**[c]	1656	8	104	50	1494	7.1 (3.1 - 13.6)	96.8 (95.8 - 97.6)	13.8 (6.1 - 25.4)	93.5 (92.2 - 94.7)
			**3rd**[d]	1542	13	87	35	1407	13.0 (7.1 - 21.2)	97.6 (96.6 - 98.3)	27.1 (15.3 - 41.8)	94.2 (92.9 - 95.3)
		**Over 2 tests**	**CTA**[e]	1731	26	104	107	1494	20.0 (13.5 - 27.9)	93.3 (92.0 - 94.5)	19.5 (13.2 - 27.3)	93.5 (92.2 - 94.7)
			**PA**[f]	1745	26	106	107	1506	19.7 (13.3 - 27.5)	93.4 (92.0 - 94.5)	19.5 (13.2 - 27.3)	93.4 (92.1 - 94.6)
		**Over 3 tests**	**CTA**[g]	1675	39	87	142	1407	31.0 (23.0 - 39.8)	90.8 (89.3 - 92.2)	21.5 (15.8 - 28.3)	94.2 (92.9 - 95.3)
			**PA**[h]	1745	39	93	142	1471	29.5 (21.9 - 38.1)	91.2 (89.7 - 92.5)	21.5 (15.8 - 28.3)	94.1 (92.8 - 95.2)
	**30**		**1st**	1745	20	112	69	1544	15.2 (9.5 - 22.4)	95.7 (94.6 - 96.7)	22.5 (14.3 - 32.6)	93.2 (91.9 - 94.4)
			**2nd**[c]	1642	11	99	56	1476	10.0 (5.1 - 17.2)	96.3 (95.3 - 97.2)	16.4 (8.5 - 27.5)	93.7 (92.4 - 94.9)
			**3rd**[d]	1520	15	81	46	1378	15.6 (9.0 - 24.5)	96.8 (95.7 - 97.6)	24.6 (14.5 - 37.3)	94.4 (93.1 - 95.6)
		**Over 2 tests**	**CTA**[e]	1731	31	99	125	1476	23.8 (16.8 - 32.1)	92.2 (90.8 - 93.5)	19.9 (13.9 - 27.0)	93.7 (92.4 - 94.9)
			**PA**[f]	1745	31	101	125	1488	23.5 (16.5 - 31.6)	92.3 (90.8 - 93.5)	19.9 (13.9 - 27.0)	93.6 (92.3 - 94.8)
		**Over 3 tests**	**CTA**[g]	1676	46	81	171	1378	36.2 (27.9 - 45.2)	89.0 (87.3 - 90.5)	21.2 (16.0 - 27.2)	94.4 (93.1 - 95.6)
			**PA**[h]	1745	46	86	171	1442	34.8 (26.8 - 43.6)	89.4 (87.8 - 90.9)	21.2 (16.0 - 27.2)	94.4 (93.1 - 95.5)
	**20**		**1st**	1745	25	107	96	1517	18.9 (12.6 - 26.7)	94.0 (92.8 - 95.2)	20.7 (13.8 - 29.0)	93.4 (92.1 - 94.6)
			**2nd**[c]	1613	13	92	76	1432	12.4 (6.8 - 20.2)	95.0 (93.7 - 96.0)	14.6 (8.0 - 23.7)	94.0 (92.6 - 95.1)
			**3rd**[d]	1473	15	74	54	1330	16.9 (9.8 - 26.3)	96.1 (94.9 - 97.1)	21.7 (12.7 - 33.3)	94.7 (93.4 - 95.8)
		**Over 2 tests**	**CTA**[e]	1734	38	92	172	1432	29.2 (21.6 - 37.8)	89.3 (87.7 - 90.7)	18.1 (13.1 - 24.0)	94.0 (92.6 - 95.1)
			**PA**[f]	1745	38	94	172	1441	28.8 (21.2 - 37.3)	89.3 (87.7 - 90.8)	18.1 (13.1 - 24.0)	93.9 (92.6 - 95.0)
		**Over 3 tests**	**CTA**[g]	1683	53	74	226	1330	41.7 (33.0 - 50.8)	85.5 (83.6 - 87.2)	19.0 (14.6 - 24.1)	94.7 (93.4 - 95.8)
			**PA**[h]	1745	53	79	226	1387	40.2 (31.7 - 49.0)	86.0 (84.2 - 87.6)	19.0 (14.6 - 24.1)	94.6 (93.3 - 95.7)
	**10**		**1st**	1745	37	95	163	1450	28.0 (20.6 - 36.5)	89.9 (88.3 - 91.3)	18.5 (13.4 - 24.6)	93.9 (92.5 - 95.0)

			**2nd** [c]	1535	17	76	111	1331	18.3 (11.0 - 27.6)	92.3 (90.8 - 93.6)	13.3 (7.9 - 20.4)	94.6 (93.3 - 95.7)
			**3rd** [d]	1362	15	58	85	1204	20.5 (12.0 - 31.6)	93.4 (91.9 - 94.7)	15.0 (8.6 - 23.5)	95.4 (94.1 - 96.5)
		**Over 2 tests**	**CTA**[e]	1735	54	76	274	1331	41.5 (33.0 - 50.5)	82.9 (81.0 - 84.7)	16.5 (12.6 - 20.9)	94.6 (93.3 - 95.7)
			**PA**[f]	1745	54	78	274	1339	40.9 (32.4 - 49.8)	83.0 (81.1 - 84.8)	16.5 (12.6 - 20.9)	94.5 (93.2 - 95.6)
		**Over 3 tests**	**CTA**[g]	1690	69	58	359	1204	54.3 (45.3 - 63.2)	77.0 (74.9 - 79.1)	16.1 (12.8 - 20.0)	95.4 (94.1 - 96.5)
			**PA**[h]	1745	69	63	359	1254	52.3 (43.4 - 61.0)	77.7 (75.6 - 79.8)	16.1 (12.8 - 20.0)	95.2 (93.9 - 96.3)

PPV: positive predictive value; NPV: negative predictive value; CI: confidence interval; TP: true positive; FN: false negative; FP: false positive; FN: false negative; CTA: cumulative test analysis; PA: programme analysis.

a. Participants who tested positive at a given threshold at year one or two were excluded from subsequent analyses.
b. Advanced adenomas were defined as adenomas ≥ 10mm, with villous or tubulovillous histology, or high grade dysplasia.
c. Includes participants who completed their second FIT, either at year two or three.
d. Includes participants who completed their third FIT.
e. Includes participants who completed at least two FITs or who tested positive at year one. Participants were classed as positive if they tested positive with either of their first two FITs.
f. Includes participants who completed at least one FIT. Participants were classed as positive if they tested positive with either of their first two FITs.
g. Includes participants who completed all three FITs or who tested positive with any FIT. Participants were classed as positive if they tested positive with any FIT.
h. Includes participants who completed at least one FIT. Participants were classed as positive if they tested positive with any FIT.

Supplementary Table 8. Sensitivity, specificity, positive predictive value (PPV), and negative predictive value (NPV) of the faecal immunochemical test (FIT) for advanced adenomas at different thresholds in participants who completed one, two, or three tests and underwent colonic examination and did not have colorectal cancer diagnosed, stratified by age at invitation date

Age at invitation date	FIT threshold (µg/g)	Test		Completed test[a]	Participants with advanced adenomas[b]		Participants without advanced adenomas[b]		Sensitivity (95% CI)	Specificity (95% CI)	PPV (95% CI)	NPV (95% CI)
				n	TP	FN	FP	TN	%	%	%	%
≤65 years	40		1st	2537	35	167	97	2238	17.3 (12.4 - 23.3)	95.8 (95.0 - 96.6)	26.5 (19.2 - 34.9)	93.1 (92.0 - 94.0)
			2nd c	2384	14	152	87	2131	8.4 (4.7 - 13.7)	96.1 (95.2 - 96.8)	13.9 (7.8 - 22.2)	93.3 (92.2 - 94.3)
			3rd d	2183	14	130	67	1972	9.7 (5.4 - 15.8)	96.7 (95.8 - 97.4)	17.3 (9.8 - 27.3)	93.8 (92.7 - 94.8)
		Over 2 tests	CTA[e]	2516	49	152	184	2131	24.4 (18.6 - 30.9)	92.1 (90.9 - 93.1)	21.0 (16.0 - 26.8)	93.3 (92.2 - 94.3)
			PA[f]	2537	49	153	184	2151	24.3 (18.5 - 30.8)	92.1 (91.0 - 93.2)	21.0 (16.0 - 26.8)	93.4 (92.3 - 94.3)
		Over 3 tests	CTA[g]	2416	63	130	251	1972	32.6 (26.1 - 39.7)	88.7 (87.3 - 90.0)	20.1 (15.8 - 24.9)	93.8 (92.7 - 94.8)
			PA[h]	2537	63	139	251	2084	31.2 (24.9 - 38.1)	89.3 (87.9 - 90.5)	20.1 (15.8 - 24.9)	93.7 (92.7 - 94.7)
	30		1st	2537	38	164	116	2219	18.8 (13.7 - 24.9)	95.0 (94.1 - 95.9)	24.7 (18.1 - 32.3)	93.1 (92.0 - 94.1)
			2nd c	2363	16	147	98	2102	9.8 (5.7 - 15.5)	95.5 (94.6 - 96.4)	14.0 (8.2 - 21.8)	93.5 (92.4 - 94.5)
			3rd d	2150	19	120	83	1928	13.7 (8.4 - 20.5)	95.9 (94.9 - 96.7)	18.6 (11.6 - 27.6)	94.1 (93.0 - 95.1)
		Over 2 tests	CTA[e]	2517	54	147	214	2102	26.9 (20.9 - 33.6)	90.8 (89.5 - 91.9)	20.1 (15.5 - 25.5)	93.5 (92.4 - 94.5)
			PA[f]	2537	54	148	214	2121	26.7 (20.8 - 33.4)	90.8 (89.6 - 92.0)	20.1 (15.5 - 25.5)	93.5 (92.4 - 94.5)
		Over 3 tests	CTA[g]	2418	73	120	297	1928	37.8 (31.0 - 45.1)	86.7 (85.2 - 88.0)	19.7 (15.8 - 24.2)	94.1 (93.0 - 95.1)
			PA[h]	2537	73	129	297	2038	36.1 (29.5 - 43.2)	87.3 (85.9 - 88.6)	19.7 (15.8 - 24.2)	94.0 (93.0 - 95.0)
	20		1st	2537	48	154	151	2184	23.8 (18.1 - 30.2)	93.5 (92.5 - 94.5)	24.1 (18.4 - 30.7)	93.4 (92.3 - 94.4)
			2nd c	2321	16	137	135	2033	10.5 (6.1 - 16.4)	93.8 (92.7 - 94.8)	10.6 (6.2 - 16.6)	93.7 (92.6 - 94.7)
			3rd d	2078	17	112	108	1841	13.2 (7.9 - 20.3)	94.5 (93.3 - 95.4)	13.6 (8.1 - 20.9)	94.3 (93.1 - 95.3)
		Over 2 tests	CTA[e]	2520	64	137	286	2033	31.8 (25.5 - 38.8)	87.7 (86.3 - 89.0)	18.3 (14.4 - 22.7)	93.7 (92.6 - 94.7)
			PA[f]	2537	64	138	286	2049	31.7 (25.3 - 38.6)	87.8 (86.4 - 89.1)	18.3 (14.4 - 22.7)	93.7 (92.6 - 94.7)
		Over 3 tests	CTA[g]	2428	81	112	394	1841	42.0 (34.9 - 49.3)	82.4 (80.7 - 83.9)	17.1 (13.8 - 20.7)	94.3 (93.1 - 95.3)
			PA[h]	2537	81	121	394	1941	40.1 (33.3 - 47.2)	83.1 (81.5 - 84.6)	17.1 (13.8 - 20.7)	94.1 (93.0 - 95.1)

	**10**		**1st**	2537	65	137	248	2087	32.2 (25.8 - 39.1)	89.4 (88.1 - 90.6)	20.8 (16.4 - 25.7)	93.8 (92.8 - 94.8)
			**2nd** [c]	2209	18	118	192	1881	13.2 (8.0 - 20.1)	90.7 (89.4 - 92.0)	8.6 (5.2 - 13.2)	94.1 (93.0 - 95.1)
			**3rd** [d]	1916	18	94	157	1647	16.1 (9.8 - 24.2)	91.3 (89.9 - 92.6)	10.3 (6.2 - 15.8)	94.6 (93.4 - 95.6)
		**Over 2 tests**	**CTA**[e]	2522	83	118	440	1881	41.3 (34.4 - 48.4)	81.0 (79.4 - 82.6)	15.9 (12.8 - 19.3)	94.1 (93.0 - 95.1)
			**PA**[f]	2537	83	119	440	1895	41.1 (34.2 - 48.2)	81.2 (79.5 - 82.7)	15.9 (12.8 - 19.3)	94.1 (93.0 - 95.1)
		**Over 3 tests**	**CTA**[g]	2439	101	94	597	1647	51.8 (44.5 - 59.0)	73.4 (71.5 - 75.2)	14.5 (11.9 - 17.3)	94.6 (93.4 - 95.6)
			**PA**[h]	2537	101	101	597	1738	50.0 (42.9 - 57.1)	74.4 (72.6 - 76.2)	14.5 (11.9 - 17.3)	94.5 (93.4 - 95.5)
**>65 years**	**40**		**1st**	2659	40	198	137	2284	16.8 (12.3 - 22.2)	94.3 (93.3 - 95.2)	22.6 (16.7 - 29.5)	92.0 (90.9 - 93.1)
			**2nd** [c]	2464	23	173	94	2174	11.7 (7.6 - 17.1)	95.9 (95.0 - 96.6)	19.7 (12.9 - 28.0)	92.6 (91.5 - 93.7)
			**3rd** [d]	2258	21	143	76	2018	12.8 (8.1 - 18.9)	96.4 (95.5 - 97.1)	21.6 (13.9 - 31.2)	93.4 (92.3 - 94.4)
		**Over 2 tests**	**CTA**[e]	2641	63	173	231	2174	26.7 (21.2 - 32.8)	90.4 (89.1 - 91.5)	21.4 (16.9 - 26.6)	92.6 (91.5 - 93.7)
			**PA**[f]	2659	63	175	231	2190	26.5 (21.0 - 32.6)	90.5 (89.2 - 91.6)	21.4 (16.9 - 26.6)	92.6 (91.5 - 93.6)
		**Over 3 tests**	**CTA**[g]	2552	84	143	307	2018	37.0 (30.7 - 43.6)	86.8 (85.4 - 88.1)	21.5 (17.5 - 25.9)	93.4 (92.3 - 94.4)
			**PA**[h]	2659	84	154	307	2114	35.3 (29.2 - 41.7)	87.3 (85.9 - 88.6)	21.5 (17.5 - 25.9)	93.2 (92.1 - 94.2)
	**30**		**1st**	2659	47	191	165	2256	19.7 (14.9 - 25.4)	93.2 (92.1 - 94.2)	22.2 (16.8 - 28.4)	92.2 (91.1 - 93.2)
			**2nd** [c]	2429	32	157	118	2122	16.9 (11.9 - 23.1)	94.7 (93.7 - 95.6)	21.3 (15.1 - 28.8)	93.1 (92.0 - 94.1)
			**3rd** [d]	2193	24	125	90	1954	16.1 (10.6 - 23.0)	95.6 (94.6 - 96.4)	21.1 (14.0 - 29.7)	94.0 (92.9 - 95.0)
		**Over 2 tests**	**CTA**[e]	2641	79	157	283	2122	33.5 (27.5 - 39.9)	88.2 (86.9 - 89.5)	21.8 (17.7 - 26.4)	93.1 (92.0 - 94.1)
			**PA**[f]	2659	79	159	283	2138	33.2 (27.2 - 39.6)	88.3 (87.0 - 89.6)	21.8 (17.7 - 26.4)	93.1 (92.0 - 94.1)
		**Over 3 tests**	**CTA**[g]	2555	103	125	373	1954	45.2 (38.6 - 51.9)	84.0 (82.4 - 85.4)	21.6 (18.0 - 25.6)	94.0 (92.9 - 95.0)
			**PA**[h]	2659	103	135	373	2048	43.3 (36.9 - 49.8)	84.6 (83.1 - 86.0)	21.6 (18.0 - 25.6)	93.8 (92.7 - 94.8)
	**20**		**1st**	2659	60	178	210	2211	25.2 (19.8 - 31.2)	91.3 (90.1 - 92.4)	22.2 (17.4 - 27.7)	92.5 (91.4 - 93.6)
			**2nd** [c]	2373	38	138	134	2063	21.6 (15.8 - 28.4)	93.9 (92.8 - 94.9)	22.1 (16.1 - 29.0)	93.7 (92.6 - 94.7)
			**3rd** [d]	2117	21	109	117	1870	16.2 (10.3 - 23.6)	94.1 (93.0 - 95.1)	15.2 (9.7 - 22.3)	94.5 (93.4 - 95.5)
		**Over 2 tests**	**CTA**[e]	2643	98	138	344	2063	41.5 (35.2 - 48.1)	85.7 (84.2 - 87.1)	22.2 (18.4 - 26.3)	93.7 (92.6 - 94.7)
			**PA**[f]	2659	98	140	344	2077	41.2 (34.9 - 47.7)	85.8 (84.3 - 87.2)	22.2 (18.4 - 26.3)	93.7 (92.6 - 94.7)
		**Over 3 tests**	**CTA**[g]	2559	119	109	461	1870	52.2 (45.5 - 58.8)	80.2 (78.5 - 81.8)	20.5 (17.3 - 24.0)	94.5 (93.4 - 95.5)
			**PA**[h]	2659	119	119	461	1960	50.0 (43.5 - 56.5)	81.0 (79.3 - 82.5)	20.5 (17.3 - 24.0)	94.3 (93.2 - 95.2)
	**10**		**1st**	2659	80	158	325	2096	33.6 (27.6 - 40.0)	86.6 (85.2 - 87.9)	19.8 (16.0 - 24.0)	93.0 (91.9 - 94.0)

			**2nd** [c]	2238	43	113	184	1898	27.6 (20.7 - 35.3)	91.2 (89.9 - 92.3)	18.9 (14.1 - 24.7)	94.4 (93.3 - 95.3)
			**3rd** [d]	1933	25	81	145	1682	23.6 (15.9 - 32.8)	92.1 (90.7 - 93.3)	14.7 (9.7 - 20.9)	95.4 (94.3 - 96.3)
		**Over 2 tests**	**CTA**[e]	2643	123	113	509	1898	52.1 (45.5 - 58.6)	78.9 (77.2 - 80.5)	19.5 (16.4 - 22.8)	94.4 (93.3 - 95.3)
			**PA**[f]	2659	123	115	509	1912	51.7 (45.1 - 58.2)	79.0 (77.3 - 80.6)	19.5 (16.4 - 22.8)	94.3 (93.2 - 95.3)
		**Over 3 tests**	**CTA**[g]	2565	148	81	654	1682	64.6 (58.1 - 70.8)	72.0 (70.1 - 73.8)	18.5 (15.8 - 21.3)	95.4 (94.3 - 96.3)
			**PA**[h]	2659	148	90	654	1767	62.2 (55.7 - 68.4)	73.0 (71.2 - 74.7)	18.5 (15.8 - 21.3)	95.2 (94.1 - 96.1)

PPV: positive predictive value; NPV: negative predictive value; CI: confidence interval; TP: true positive; FN: false negative; FP: false positive; FN: false negative; CTA: cumulative test analysis; PA: programme analysis.

a. Participants who tested positive at a given threshold at year one or two were excluded from subsequent analyses.
b. Advanced adenomas were defined as adenomas ≥ 10mm, with villous or tubulovillous histology, or high grade dysplasia.
c. Includes participants who completed their second FIT, either at year two or three.
d. Includes participants who completed their third FIT.
e. Includes participants who completed at least two FITs or who tested positive at year one. Participants were classed as positive if they tested positive with either of their first two FITs.
f. Includes participants who completed at least one FIT. Participants were classed as positive if they tested positive with either of their first two FITs.
g. Includes participants who completed all three FITs or who tested positive with any FIT. Participants were classed as positive if they tested positive with any FIT.
h. Includes participants who completed at least one FIT. Participants were classed as positive if they tested positive with any FIT.

ECONOMIC ANALYSIS OF FAECAL IMMUNOCHEMICAL TESTS (FIT) VERSUS COLONOSCOPY SURVEILLANCE

INTRODUCTION

FIT has the potential to reduce costs associated with post-polypectomy surveillance of intermediate-risk patients. The aim of the economic evaluation was to undertake a cost and cost-effectiveness analysis, comparing costs and outcomes of three annual FITs versus colonoscopy surveillance at three years.

METHODS

For the cost analysis, we calculated costs of annual FIT surveillance and colonoscopy surveillance at three years. For the cost-effectiveness analysis, outcomes were the number of AAs and CRCs detected by each surveillance regimen. We expressed cost-effectiveness as the incremental cost per additional AA and CRC detected by colonoscopy versus FIT surveillance.

The analysis was undertaken from the perspective of the UK National Health Service (NHS).[1] Costs were expressed in 2015 pounds sterling and inflated where necessary.[2] The time horizon was chosen to be three years, to match the three year cycle time of colonoscopy surveillance. Costs were discounted at a rate of 3.5% for every year after year one.

A full cost-utility analysis, assessing costs and quality-adjusted life years (QALYs) over a lifetime horizon, was not conducted because this would have required separate data for the outcomes and treatment pathways associated with each surveillance regimen.

There were no missing data for the analyses.

Generating a control arm

The ‘FIT for Follow-Up’ study was a single-arm study and as such, we had to generate a pseudo-control arm to estimate the cost and outcomes of three-yearly colonoscopy surveillance in the absence of FIT.

We created this pseudo-control arm based on the assumption that participants who ‘did not attend’ (DNA) their FIT-positive colonoscopies would also have been DNA participants for the routine three-year colonoscopy. Similarly we assumed that participants who were lost to follow-up during the study would have failed to attend the routine three-year colonoscopy. Therefore, the size of the pseudo-control arm was estimated by taking the intervention arm, the 5938 participants who consented and returned a FIT at year one, and subtracting 61 DNA participants for FIT-positive colonoscopies, 519 DNA participants for the routine three-year colonoscopy, and 133 participants who were lost to follow-up. This left 5225 participants in the pseudo-control arm.

Supplementary Figure 1 shows the number of participants in the FIT arm who completed each FIT, the estimated number of participants in the pseudo-control arm who would have attended three-yearly colonoscopy in the absence of FIT, and the number of colonoscopies, number of AAs and CRCs detected, and costs incurred in each arm.

Resource use and costs

We restricted costs to surveillance costs and did not include costs of treating observed cases of disease. We included costs of FIT kits,[3] diagnostic procedures undertaken as a result of positive FIT results, polypectomy, and treatment of colonoscopy complications (i.e. bowel perforation and gastrointestinal bleeding) (Supplementary Table 9). Unit costs for diagnostic procedures were taken from the National Schedule of Reference Costs for 2014-15.[4] The probability of bowel perforation occurring during colonoscopy was assumed to be 0.0017 without polypectomy and 0.0008 with polypectomy,[5] and the probability of gastrointestinal bleeding occurring after colonoscopy was assumed to be 0.00439.[5] Unit costs for treatment of these complications were also taken from the National Schedule of Reference Costs (Supplementary Table 9).[4] When AAs were detected, procedures were costed with polypectomy, as it was assumed that AAs would be removed during colonoscopy. For the base case analysis, DNA participants were assumed to incur no cost.

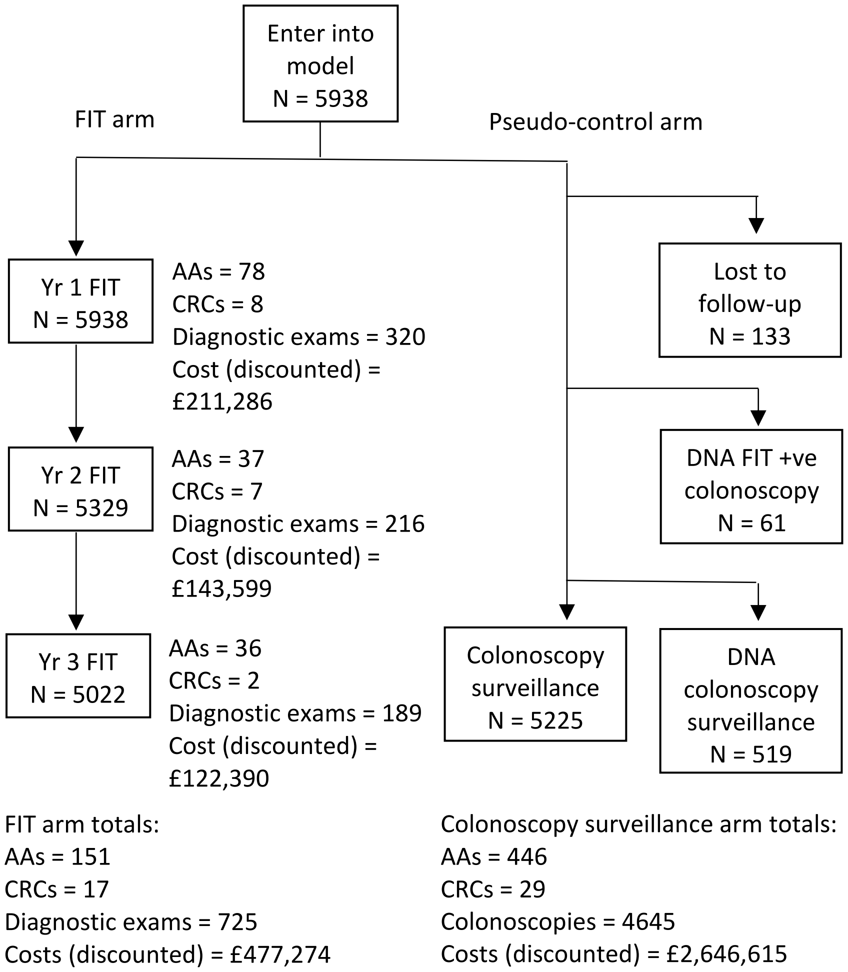

FIT: faecal immunochemical test; AAs: advanced adenomas; CRC: colorectal cancer; DNA: 'did not attend' participants

FIT arm data was drawn from the 'FIT for Follow-Up' study CONSORT diagram (Figure 1). The pseudo-control arm estimates the number of participants who would have undergone three-yearly colonoscopy surveillance in the absence of the 'FIT for Follow-Up' study.

Supplementary Figure 1. Estimated costs and outcomes associated with annual faecal immunochemical test (FIT) surveillance and three-yearly colonoscopy surveillance

Outcomes

Outcomes were the number of AAs and CRCs detected in each arm of the model.

Cost-effectiveness analysis

We calculated total costs and mean costs per participant of each surveillance regimen. From these figures, we calculated total cost differences and incremental costs per participant of colonoscopy versus FIT surveillance.

Since FIT surveillance was less costly and less effective than colonoscopy surveillance, incremental cost-effectiveness ratios (ICERs) were calculated in terms of the incremental cost per additional AA and CRC detected by colonoscopy versus FIT surveillance. We calculated these by subtracting the total cost of FIT surveillance from the total cost of colonoscopy surveillance, and dividing by the number of AAs (or CRCs) detected by colonoscopy surveillance minus the AAs (or CRCs) detected by FIT surveillance. As the numerators of the ICERs (incremental costs) and denominators (differences in numbers of AAs and CRCs detected) were both positive, ICERs were positive, and higher positive values reflected worse value for money of colonoscopy versus FIT surveillance, and therefore better value for money of FIT versus colonoscopy surveillance.

Supplementary Table 9. Unit costs

Parameters	Value	Source
FIT kit returned	£5.14	Murphy et al, 2015 [3]
FIT kit not returned	£1.66	Murphy et al, 2015 [3]
Colonoscopy	£519.42	National schedule of reference costs 2014/15, diagnostic, 19 years and over [4]
Colonoscopy with polypectomy	£601.86	National schedule of reference costs 2014/15, therapeutic, 19 years and over [4]
Computed tomography colonography	£87.92	National schedule of reference costs 2014/15, diagnostic imaging [4]
Diagnostic flexible sigmoidoscopy	£381.61	National schedule of reference costs 2014/15, diagnostic, 19 years and over [4]
Flexible sigmoidoscopy with polypectomy	£480.76	National schedule of reference costs 2014/15, therapeutic, 19 years and over [4]
Cost of treating bowel perforation	£5911.08	National schedule of reference costs 2014/15, major large intestine procedure, 19 years and over [4]
Cost of treating gastrointestinal bleed	£2498.14	National schedule of reference costs 2014/15, gastrointestinal bleed with single intervention [4]
Probability of bowel perforation during colonoscopy without polypectomy	0.0008	Atkin et al, 2002 [5]
Probability of bowel perforation during colonoscopy with polypectomy	0.0017	Atkin et al, 2002 [5]
Probability of gastrointestinal bleeding after colonoscopy	0.00439	Atkin et al, 2002 [5]

FIT: faecal immunochemical test

Sensitivity analyses

We conducted a probabilistic sensitivity analysis,[1] varying the following parameters:

- Number of FITs kits returned at each year
- Number of colonoscopies required at each year
- Number of DNA participants at each year
- Number of AAs and CRCs detected by FIT at each year
- Unit costs of colonoscopy and treatment of colonoscopy complications

We used probabilities to characterise the number of returned FITs, colonoscopies, DNAs, detected AAs and CRCs, and modelled uncertainty in these probabilities using Beta distributions.[6] We modelled uncertainty in unit costs using uniform distributions, allowing the values to vary randomly from the base case value by ±25%.

For each simulation of the probabilistic sensitivity analysis, a random value was selected from the distribution of each parameter, and used to estimate the mean cost and mean number of detected AAs and CRCs associated with FIT surveillance. These estimates were used to calculate the incremental costs, differences in numbers of detected AAs and CRCs, and ICERs for colonoscopy versus FIT surveillance. A total of 5000 simulations were run, yielding 5000 separate sets of results. We calculated 95% uncertainty intervals (UIs) as the 2.5th and 97.5th percentiles of the 5000 simulated values.

A deterministic sensitivity analysis was also conducted. Using data on the performance of FIT at various positivity thresholds from the main study, we explored the effect on cost and cost-effectiveness of using these different thresholds. A threshold of 40µg/g was applied in the base case analysis and thresholds of 30µg/g, 20µg/g, and 10µg/g were considered in sensitivity analyses. The numbers of AAs and CRCs that would have been detected using the different thresholds were estimated based on the diagnostic accuracy estimates reported in the main study. We assumed a linear relationship between the number of FIT positive results and overall costs associated with FIT surveillance, on the basis that more patients would be classed as positive and thus referred for colonic examination at lower thresholds.

We also explored the impact of varying diagnostic procedure unit costs using deterministic sensitivity analysis. First we assumed that all procedures were undertaken as elective inpatient procedures. We then assumed that all procedures were outpatient procedures. We used weighted averages of the upper and lower quartiles of the unit cost of computed tomography colonography. We varied the unit costs of FIT kits and treatment of colonoscopy complications by ±25%. To examine the impact of varying the cost of DNA participants, we valued them at the full cost of colonoscopy without polypectomy, rather than at zero cost as in the base case analysis.

Budget impact

The budget impact of replacing colonoscopy surveillance with FIT surveillance nationally over a screening cycle was estimated by multiplying the incremental costs per participant by the total number of estimated eligible participants for one cycle. Our estimate was calculated assuming that approximately 4.5 million people aged 60–74 years would be screened adequately for CRC in England

over a 2.5 year cycle;[7] 2% of these would have an abnormal result at screening and be offered a colonic examination;[8] attendance at colonic examination would be 88%;[9] and 16% of those undergoing colonic examination would be classed as being at intermediate-risk.[10]

RESULTS

Costs and outcomes

The total cost of annual FIT surveillance over a three-year cycle was estimated to be £477,274 at a threshold of 40µg/g, and the cost of colonoscopy surveillance at three years was estimated to be £2,646,615 (Supplementary Table 10). FIT surveillance therefore produced a cost saving of £2,169,341 compared with colonoscopy surveillance.

Supplementary Table 10. Costs and outcomes of colonoscopy and faecal immunochemical test (FIT) surveillance at various FIT thresholds

Costs and outcomes	Three-yearly colonoscopy	Annual FIT (40µg/g)	Annual FIT (30µg/g)	Annual FIT (20µg/g)	Annual FIT (10µg/g)
Absolute cost	£2,646,615	£477,274	£562,538	£685,383	£944,957
Cost per participant	£446	£80	£95	£115	£159
AAs detected	446	151	182	205	254
CRCs detected	29	17	19	21	22

AA: advanced adenoma; CRC: colorectal cancer; FIT: faecal immunochemical test.

The mean total cost per participant for FIT surveillance was £80 at 40µg/g and £159 at 10µg/g, compared to £446 for colonoscopy surveillance (Supplementary Table 10). For FIT surveillance, most of the total cost (80%) was accounted for by colonoscopies undertaken following a positive FIT; the remainder was split between the cost of FIT kits, treatment of colonoscopy complications, and alternative diagnostic tests (computed tomography colonography and flexible sigmoidoscopy). The cost of colonoscopy surveillance was completely accounted for by colonoscopies undertaken.

FIT surveillance detected fewer AAs than colonoscopy surveillance (151 at 40µg/g versus 446), and fewer CRCs (17 at 40µg/g versus 29) (Supplementary Table 10).

Cost-effectiveness analysis

At a threshold of 40µg/g, the mean incremental cost per participant for colonoscopy versus FIT surveillance was £365 (95% UI £327 to £390), and colonoscopy surveillance detected 295 more AAs (95% UI 273 to 316) and twelve more CRCs (95% UI 3 to 19) than FIT surveillance (Supplementary Table 11). This shows that FIT surveillance was cheaper and less effective at detecting AAs and CRCs than colonoscopy surveillance. The incremental cost-effectiveness of colonoscopy versus FIT surveillance was £7354 (95% UI £6468 to £8155) per additional AA detected, and £180,778 (95% UI £111,913 to £618,140) per additional CRC detected (Supplementary Table 11).

Sensitivity analysis of faecal immunochemical test (FIT) threshold

In our sensitivity analysis that looked at lower FIT thresholds, FIT surveillance was again cheaper and less effective at detecting AAs than colonoscopy surveillance (Supplementary Tables 10 and 11). However, at lower thresholds, there is a chance that FIT surveillance was not less effective at detecting

CRCs. For example, whilst we estimated that colonoscopy surveillance detected seven more CRCs than FIT at 10µg/g, the UI crossed zero (95% UI -3 to 15) (Supplementary Table 11). At lower thresholds, the differences in total costs and numbers of AAs and CRCs detected by FIT and colonoscopy surveillance were smaller. The ICER values increased, representing better value for money of FIT versus colonoscopy surveillance. At 10µg/g, the incremental cost-effectiveness of colonoscopy versus FIT surveillance was £8863 (95% UI £7018 to £10,939) per additional AA detected, and £243,094 (95% UI -£1,242,531 to £1,990,865) per additional CRC detected (Supplementary Table 11).

Sensitivity analysis of the cost of 'did not attend' (DNA) participants

When DNA participants were valued at the full cost of colonoscopy without polypectomy, the incremental cost per participant for colonoscopy versus FIT surveillance was £407. The incremental cost-effectiveness of colonoscopy versus FIT surveillance was £8198 per additional AA detected, and £201,522 per additional CRC detected (Supplementary Table 12).

Sensitivity analysis of diagnostic procedure costs

Incremental costs and cost-effectiveness estimates were largely insensitive to changes in unit costs. The cost of colonoscopies had the greatest impact on the analysis (Supplementary Table 12).

Budget impact analysis

As FIT surveillance was less costly than colonoscopy surveillance, the budget impact from replacing colonoscopy surveillance with FIT surveillance nationally would be -£4.7 million (95% UI -£5.0 million to -£4.2 million). This calculation is based on our estimation that 12,777 individuals would be eligible for surveillance, and that FIT surveillance would produce a cost saving per participant of -£365 (95% UI -£390 to -£327) at 40µg/g. At 30µg/g, 20µg/g, and 10µg/g, the cost savings per participant for FIT versus colonoscopy surveillance would be -£351 (95% UI -£377 to -£309), -£330 (95% UI -£361 to -£284), and -£287 (95% UI -£326 to -£237), respectively, and the budget impact would be -£4.5 million (95% UI -£4.8 million to -£4.0 million), -£4.2 million (95% UI -£4.6 million to -£3.6 million), and -£3.7 million (95% UI -£4.2 million to -£3.0 million), respectively.

DISCUSSION

This economic analysis has demonstrated that annual FIT surveillance is cheaper than three-yearly colonoscopy surveillance, but less effective at detecting AAs and CRCs. Our sensitivity analyses suggested that the results were most sensitive to the FIT positivity threshold, cost of DNAs, and cost of colonoscopies. In all cases, FIT surveillance was less costly than colonoscopy surveillance. At lower FIT thresholds, the incremental cost per additional AA and CRC detected by colonoscopy versus FIT surveillance was significantly higher. This is because the higher diagnostic costs associated with FIT surveillance at the lower thresholds (due to greater referral rates for colonic examination) were offset by the increase in number of AAs and CRCs detected. Therefore, if three-yearly colonoscopy surveillance were to be replaced with annual FIT, the most cost-effective strategy would be to adopt a low FIT threshold. This would minimise the risk of missing AAs and CRCs, whilst still producing significant cost savings.

Our economic analysis has a number of limitations. Firstly, as the analysis was based on a single-arm trial, we created a pseudo-control arm to evaluate the cost and outcomes of people who would have

had routine three-yearly colonoscopy surveillance in the absence of FIT. This is a suboptimal alternative to using data from a real control arm in a randomised controlled trial.

Secondly, our analysis only included the short-term costs and outcomes associated with FIT and colonoscopy surveillance. We did not consider the long-term costs and outcomes associated with missed AAs and CRCs and so were unable to estimate lifetime costs and QALYs associated with each surveillance regimen. Our results are therefore underestimations of the true costs of both surveillance regimens. Given the potential cost implications of missed CRCs, this underestimation might be especially pronounced for FIT surveillance.

In the light of these limitations, we recommend that future studies evaluate annual FIT versus three-yearly colonoscopy surveillance in terms of incremental costs per QALYs gained over a lifetime horizon. Ideally, analyses would be based on data from randomised controlled trials, enabling treatment and control arms to be directly compared and removing the need to create a pseudo-control arm. Such analyses would account for surveillance costs, costs of treating diagnosed and missed CRCs, as well as the impact of each surveillance regimen on health-related quality of life.

Supplementary Table 11. Economic evaluation of colonoscopy versus faecal immunochemical test (FIT) surveillance at various FIT thresholds

**Output parameters**	**Colonoscopy versus FIT surveillance at various FIT thresholds**			
	**40μg/g**	**30μg/g**	**20μg/g**	**10μg/g**
Total cost difference	£2,169,341 (£1,943,356 to £2,313,530)	£2,084,077 (£1,832,576 to £2,240,934)	£1,961,233 (£1,689,300 to £2,145,814)	£1,701,658 (£1,408,908 to £1,937,336)
Incremental cost per participant	£365 (£327 to £390)	£351 (£309 to £377)	£330 (£284 to £361)	£287 (£237 to £326)
Difference in number of detected AAs	295 (273 to 316)	264 (241 to 287)	241 (215 to 266)	192 (164 to 221)
Difference in number of detected CRCs	12 (3 to 19)	10 (1 to 17)	8 (-2 to 15)	7 (-3 to 15)
Incremental cost per additional AA detected	£7354 (£6468 to £8155)	£7894 (£6816 to £8928)	£8138 (£6826 to £9435)	£8863 (£7018 to £10,939)
Incremental cost per additional CRC detected	£180,778 (£111,913 to £618,140)	£208,408 (£105,523 to £952,847)	£245,154 (-£1,116,127 to £1,670,487)	£243,094 (-£1,242,531 to £1,990,865)

AA: advanced adenoma; CRC: colorectal cancer; FIT: faecal immunochemical test.

Figures in parentheses are 95% uncertainty intervals, calculated as the 2.5th and 97.5th percentiles of the simulated values.

Supplementary Table 12. Sensitivity analysis of the cost of 'did not attend' (DNA) participants and diagnostic procedure unit costs

Input parameters	Incremental cost per participant	Incremental cost per additional AA detected	Incremental cost per additional CRC detected
Base case	£365	£7354	£180,778
DNAs valued at full cost	£407	£8198	£201,522
Low cost colonoscopy	£266	£5949	£146,238
Low cost colonoscopy with polypectomy	£345	£6954	£170,963
Low cost flexible sigmoidoscopy	£366	£7366	£181,075
Low cost computed tomography colonography	£365	£7356	£160,786
Low cost of treating bowel perforation	£364	£7336	£180,336
Low cost of treating gastrointestinal bleed	£363	£7315	£179,822
FIT kit cost -25%	£362	£7285	£179,078
High cost colonoscopy	£578	£11,641	£286,180
High cost colonoscopy with polypectomy	£382	£7691	£189,066
High cost flexible sigmoidoscopy	£365	£7354	£180,787
High cost computed tomography colonography	£366	£7372	£181,220
High cost of treating bowel perforation	£367	£7393	£181,734
High cost of treating gastrointestinal bleed	£363	£7421	£269,939
FIT kit cost +25%	£362	£7285	£179,078

AA: advanced adenoma; CRC: colorectal cancer; DNA: 'did not attend' participants; FIT: faecal immunochemical test

REFERENCES

1. National Institute for Health and Care Excellence (NICE). Guide to the methods of technology appraisal 2013. London: NICE; 2013. [Available from: https://www.nice.org.uk/guidance/pmg9/resources/guide-to-the-methods-of-technology-appraisal-2013-pdf-2007975843781 (accessed June 2018).

2. Curtis L, Burns A. Unit Costs of Health and Social Care 2015. The University of Kent, Canterbury: Personal Social Services Research Unit;2015. [Available from: https://www.pssru.ac.uk/pub/uc/uc2015/full.pdf (accessed June 2018).

3. Murphy J, Gray A. The cost-effectiveness of immunochemical faecal occult blood testing vs. guaiac faecal occult blood testing for colorectal cancer screening in the NHS Bowel Cancer Screening Programme: Report to the UK National Screening Committee. Health Economics Research Centre, University of Oxford, 2015.

4. Department of Health and Social Care. National Schedule of Reference Costs 2014–2015. London: Department of Health and Social Care; 2015. [Available from: https://www.gov.uk/government/publications/nhs-reference-costs-2014-to-2015 (accessed June 2018).

5. Atkin WS, Cook CF, Cuzick J, et al. Single flexible sigmoidoscopy screening to prevent colorectal cancer: baseline findings of a UK multicentre randomised trial. *Lancet*. 2002;359(9314):1291-300.

6. Briggs A, Claxton K, Sculpher M. Decision Modelling for Health Economic Evaluation. New York: Oxford University Press; 2006.

7. National Health Service (NHS) England Data Catalogue. Cancer Screening Coverage – Bowel Cancer. London: Public Health England; 2016. [Available from: https://data.england.nhs.uk/dataset/phe-indicator-91720 (accessed June 2018).

8. Cancer Research UK, National Health Service (NHS) Bowel Cancer Screening Programme. Bowel Cancer Screening: The Facts. [Available from: https://www.gov.uk/government/uploads/system/uploads/attachment_data/file/598271/BOSP01_bowel_cancer_facts.pdf (accessed June 2018). London: Public Health England; 2016.

9. Morris S, Baio G, Kendall E, et al. Socioeconomic variation in uptake of colonoscopy following a positive faecal occult blood test result: a retrospective analysis of the NHS Bowel Cancer Screening Programme. *Br J Cancer*. 2012;107(5):765-71.

10. Seaman H. National Health Service (NHS) Bowel Cancer Screening Programme Southern Programme Hub Annual Report 2014/15. [Available from: http://www.royalsurrey.nhs.uk/wp-content/uploads/2015/12/BCSP-Southern-Hub-Annual-Report-2014-2015.pdf (accessed June 2018).
